# An Expressed Sequence Tag collection from the male antennae of the Noctuid moth *Spodoptera littoralis*: a resource for olfactory and pheromone detection research

**DOI:** 10.1186/1471-2164-12-86

**Published:** 2011-01-29

**Authors:** Fabrice Legeai, Sébastien Malpel, Nicolas Montagné, Christelle Monsempes, François Cousserans, Christine Merlin, Marie-Christine François, Martine Maïbèche-Coisné, Frédérick Gavory, Julie Poulain, Emmanuelle Jacquin-Joly

**Affiliations:** 1 IRISA, équipe Symbiose, Campus universitaire de Beaulieu, 35042 Rennes Cedex, France; 2 INRA, UMR-A 1272 INRA-UPMC PISC Physiologie de l'Insecte: Signalisation et Communication, route de Saint-Cyr, 78026 Versailles Cedex, France; 3 INRA, UMR 1231 INRA-Université Montpellier II BIVI, Place Eugène Bataillon, 34095 Montpellier Cedex 5, France; 4 Genoscope, Centre National de Séquençage, 2 rue Gaston Crémieux, BP 191, F-91057 Evry Cedex, France; 5 UMR 5548, University of Bourgogne, 6 bd Gabriel, F-21000 Dijon, France; 6 Department of Neurobiology, University of Massachusetts Medical School, 01604, Worcester, MA, USA

## Abstract

**Background:**

Nocturnal insects such as moths are ideal models to study the molecular bases of olfaction that they use, among examples, for the detection of mating partners and host plants. Knowing how an odour generates a neuronal signal in insect antennae is crucial for understanding the physiological bases of olfaction, and also could lead to the identification of original targets for the development of olfactory-based control strategies against herbivorous moth pests. Here, we describe an Expressed Sequence Tag (EST) project to characterize the antennal transcriptome of the noctuid pest model, *Spodoptera littoralis*, and to identify candidate genes involved in odour/pheromone detection.

**Results:**

By targeting cDNAs from male antennae, we biased gene discovery towards genes potentially involved in male olfaction, including pheromone reception. A total of 20760 ESTs were obtained from a normalized library and were assembled in 9033 unigenes. 6530 were annotated based on BLAST analyses and gene prediction software identified 6738 ORFs. The unigenes were compared to the *Bombyx mori *proteome and to ESTs derived from Lepidoptera transcriptome projects. We identified a large number of candidate genes involved in odour and pheromone detection and turnover, including 31 candidate chemosensory receptor genes, but also genes potentially involved in olfactory modulation.

**Conclusions:**

Our project has generated a large collection of antennal transcripts from a Lepidoptera. The normalization process, allowing enrichment in low abundant genes, proved to be particularly relevant to identify chemosensory receptors in a species for which no genomic data are available. Our results also suggest that olfactory modulation can take place at the level of the antennae itself. These EST resources will be invaluable for exploring the mechanisms of olfaction and pheromone detection in *S. littoralis*, and for ultimately identifying original targets to fight against moth herbivorous pests.

## Background

Olfaction serves to detect environmental chemical information. Nocturnal insects such as moths appear as ideal models to study the physiology of olfaction, since this sensory modality is essential for their survival and thus highly developed. In particular, the moth pheromone detection system is extremely sensitive: a male can smell and locate a female miles away for mating [1]. It has been for long an established model to study the molecular bases of olfaction [2]. In addition, moths include diverse and important pests of crops, forests and stored products. Olfaction underlies several behaviours critical for crop aggression, including sex pheromone-mediated reproduction, host selection and oviposition [3]. It is thus an attractive target for pest control. For example, several olfactory-based strategies have been developed to control moth populations, such as mass trapping and mating disruption [4]. Better knowledge on the molecular mechanisms by which an odour generates a neuronal signal could lead to the identification of targets for the development of new safe control strategies.

The olfactory signals are detected by the antennae, the peripheral olfactory organs, where they are transformed in an electrical signal that will be further integrated in the central nervous system. Located on the head, the antennae carry thousands of innervated olfactory structures, the sensilla, which house the olfactory receptor neurons. Within these sensilla, odour recognition relies on the expression of a diversity of olfactory genes involved in different steps (reviewed in [5]). First, volatile odours are bound by odorant-binding proteins (OBPs) in order to cross the aqueous sensillum lymph that embeds the olfactory neuron dendrites. The OBP family notably includes two sub-families: the pheromone-binding proteins (PBPs), thought to transport pheromone molecules, and the general odorant-binding proteins (GOBPs), thought to transport general odorants such as plant volatiles [6,7]. Many other soluble secreted proteins are also found in abundance within the sensillum lymph, examples are the so-called chemosensory proteins (CSPs), the antennal binding proteins X (ABPX) and the sensory appendage proteins (SAPs) [8], but their role in olfaction remains elusive. After crossing the lymph, odorant molecules interact with olfactory receptors (ORs, called pheromone receptors or PRs when ligands are pheromones) located in the dendritic membrane of receptor neurons (reviewed in [9]). The chemical signal is then transformed into an electric signal that will be transmitted to the brain. Sensory neuron membrane proteins (SNMPs), located in the dendritic membrane of pheromone sensitive neurons [7,10], are thought to trigger ligand delivery to the receptor [11]. Signal termination may then be ensured by specific enzymes, the odorant-degrading enzymes (ODEs, called pheromone-degrading enzymes or PDEs when substrates consist of pheromones) (reviewed in [7]). Although we still lack a consensus on the exact function of each protein family, the occurrence of a large diversity within these families suggests they participate in the specificity of odour recognition [2]. The combinatorial expression of these proteins within a sensillum may ensure the specificity and the sensitivity of the olfactory reception, defining the functional phenotypes of olfactory receptor neurons.

Complete or partial repertoires of putative olfactory genes have been established in insect species with an available sequenced genome. In other species for which no genomic data are yet available, such as crop pest moths, we still lack a global view of the olfactory genes. Homology-based cloning strategies led to the identification of conserved genes, such as OBPs [12], but failed to reliably identify divergent genes, in particular ORs. Insect ORs constitute an atypical family of seven transmembrane domain receptors exhibiting a pronounced intra - as well as inter-specific sequence diversity. As a result, OR repertoires have been established using the complete or partial genome databases of, among examples, the dipterans *Drosophila melanogaster *[13-15] and *Anopheles gambiae *[16], the hymenopterans *Apis mellifera *[17] and *Nasonia vitripennis *[18], the coleopteran *Tribolium castaneum *[19] and the lepidopteran *B. mori *[20,21]. In other Lepidoptera, only few ORs and PRs have been identified to date [22-27]. Among them, one atypical subtype of ORs, defining the so-called *D. melanogaster *OR83b orthologue family, is required for the functionality of the other ORs [28,29]. This subtype is highly conserved among insects and orthologues have been identified in numerous species, including a variety of moths [30,31]. The identification of additional moth ORs and PRs is thus challenging. This will provide information on the evolution and diversification of this receptor family in this biodiverse group of insects and, in a context of plant protection, ORs appear as good targets for the design of molecules capable to interfere with the ligand and thus the receptor response and the associated insect behaviour.

Expressed Sequence Tag (EST) sequencing strategies are efficient in identifying a large number of genes expressed in a particular tissue, thus providing information on the physiological properties of this specific tissue. Such approaches are particularly relevant when no genomic data are available for the target species. EST collections are now established for various tissues in several Lepidoptera species, especially in *B. mori*, the only Lepidoptera for which the genome has been sequenced [32]. However, only two EST strategies have been previously engaged on antennae. In 1999, Robertson et al [33] sequenced 300 ESTs from *Manduca sexta *antennae and identified a variety of candidate OBPs, but no ORs. In 2008, Jordan et al [34] sequenced 5739 ESTs from the antennae of the tortricid, *Epiphyas postvittana*, whose analysis revealed members of families implicated in odorant and pheromone binding (PBPs, GOBPs, ABPXs, CSPs) and turnover (putative ODEs). Only three genes encoding putative ORs were found, including one encoding an orthologue of the non-canonical odorant receptor OR83b from *Drosophila*.

In view of these difficulties in identifying ORs, we combined high-throughput sequencing and normalization of a cDNA library, prepared from the antennae of the cotton leafworm *Spodoptera littoralis*. This polyphagous noctuid species is one of the major pests of cotton, and much is known about its olfaction, thanks to previous behavioural and electrophysiological investigations: the sex pheromone, plant volatiles activating olfactory neurons, and various functional types of olfactory sensilla have been characterized [35]. *S. littoralis *thus appears particularly well-suited to establish the molecular bases of olfactory and pheromone reception in a crop pest from the noctuid family, which groups some of the most aggressive herbivorous pests.

In this paper, we report the analysis and annotation of 20760 ESTs obtained from *S. littoralis *male antennae. First, this allowed us to establish the use of transcriptome sequencing to identify putative olfactory genes, and among them chemosensory receptor-encoding genes. We report on the identification of 31 candidate olfactory/gustatory receptor genes in a species for which no genomic data are available. Second, we provide evidence that the antennae express different non olfactory genes possibly involved in processes such as defense, plasticity and circadian rhythms. These EST resources will be invaluable for exploring the mechanism of olfaction and pheromone detection, but also other antennal processes, in a pest model species.

## Results and discussion

### EST statistics and unigene prediction

A total of 20760 ESTs (mean length: 958.1 bp, median length: 820 bp, max length: 1525 bp, min length: 40 bp, table 1) were obtained from male antennae of *S. littoralis*. A first batch of 2211 sequences, obtained by Genome-express (Grenoble, France), has been deposited in the GenBank database [GenBank:GW824594-GW826804]. A second batch of 18549 ESTs, sequenced by the Genoscope (Evry, France), has been deposited in the European Molecular Biology Laboratory (EMBL) [EMBL:FQ014236-FQ032656,] and GenBank [GenBank:HO118288-HO118415]. Full description of all ESTs is available in additional file 1. The ESTs were processed and assembled. 14385 ESTs (69.3%, mean length: 929.5 bp, median length: 804 bp) were assembled into 2705 contigs, 6328 sequences corresponded to singletons and 47 were removed due to vector contamination. The contigs have been submitted to INSDC into the TSA division [EZ980986-EZ983690]. All together, singletons and contigs were merged into a set of 9033 unigenes that putatively represent different transcripts (mean length: 1095.2 bp, median length: 1198 bp, max length: 3609 bp, min length: 40 bp, table 1). Full description of the unigenes is available in additional file 2. On average, each contig was assembled from 5.32 ESTs. 6328 (~70%) of the unigenes were singletons, 899 (33.23%) of contig sequences had two ESTs, 481 (17.78%) had three ESTs, and 275 (10.20%) had more than 10 ESTs. It has to be pointed out that we did only 5' end sequencing that, together with splice variants, polymorphism or reverse transcriptase errors, may have led to under-assembly and thus over-estimation of unigene counts. Examples of such under-assembly, revealed by manual OBP and OR analyses, are discussed later.

**Table 1:** Data summary

	Counts (total nb)	Min. length (bp/aa)	Average length (bp/aa)	Max length (bp/aa)	Median lenght (bp/aa)	Accession numbers
All ESTs	20760	40	958.1	1525	820	FQ0142366-FQ032656
						GW824594-GW826804
						HO118288-HO118415

Singletons	6328	40	1029.5	1500	1172	

Contigs	2705	40	1277.1	3609	1290	EZ980986-EZ983690

All unigenes	9033	40	1095.2	3609	1198	

Annotated unigenes*	6530	40	1127.9	3609	1217	

Predicted peptides	6738	30	215.14	922	221	

* unigenes with a significant match against nr, *Drosophila melanogaster *proteome or *Bombyx mori *transcripts.

### Identification of putative ORFs

**Figure 1:**
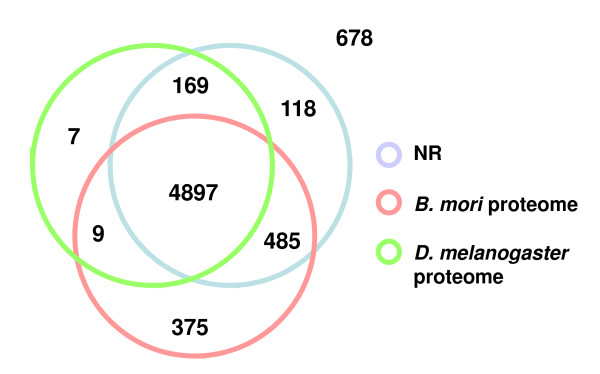
**Number of proteins translated from *S. littoralis *antennal ORFs overlapping with the GenBank non-redundant protein database (NR)**. (7 686 184 entries, July 2009), the *B. mori *proteome (14 632 entries, SilkDB April 2008 release) and the *D. melanogaster *proteome (21 647 entries, FlyBase release v5.16) (e-value cut off: 1e-5).

Among the 9033 unigenes, 6738 presented a coding region (74.6%, mean length: 215.14 aa, median length: 221 aa, max length: 922 aa, min length: 30 aa, table 1). Protein sequences translated from the predicted open reading frame (ORF) set were compared to the non-redundant protein database (NR) and to the *D. melanogaster *and *B. mori *complete proteomes (e-value cut off: 1e-5) (Figure 1). Most of the sequences (90%) translated from predicted ORFs, showed similarity to known proteins. 678 ORFs presented no similarity at all. The 972 protein sequences having no similarity with the *B. mori *proteome were further compared to the *B. mori *genome using TBLASTX (e-value cut off: 1e-20), since the *B. mori *protein prediction available in SilkDB may have missed some genes. 713 remaining *S. littoralis *protein sequences had no similarity with any *B. mori *gene. 50 were classified in a gene ontology term and were analyzed using BLAST2GO (Additional file 3). Interestingly, we found enrichment in putative proteins involved in defense response to bacteria (FDR: 7,21E-004), antifungal humoral response (2,04E-006), xenobiotic metabolism processes (8,08E-006) and interaction between organisms (1,56E-007). An enrichment in defense-related objects was recently observed by Vogel et al [36] in the transcriptome of the noctuid *Heliothis virescens *pheromone glands, when compared to that of *B. mori*.

The 678 sequences presenting no similarity with any known protein were analyzed using Interproscan [37] (Additional file 4). Interestingly, a sequence of this set presented a PBP/GOBP protein domain and appeared as a new original candidate OBP, in addition to the others we discovered (see paragraph below).

### Specificity analysis using ESTs

**Figure 2:**
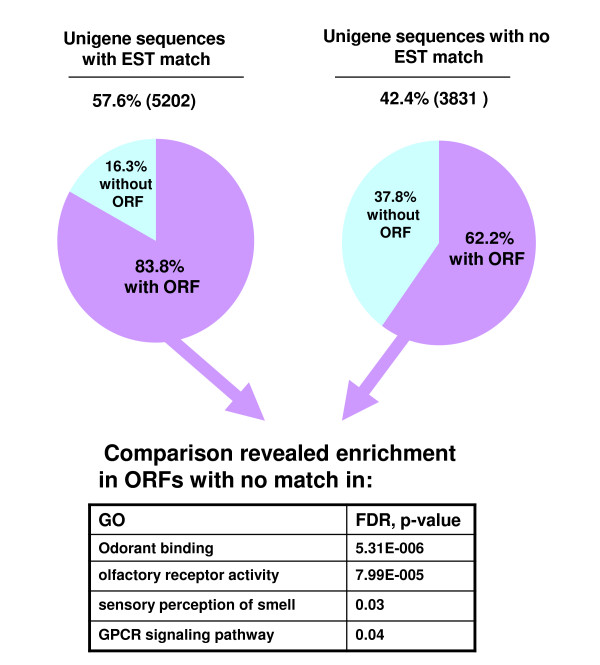
**Distribution of *S. littoralis *ESTs with or without match in NCBI EST database using BLASTN (e-value cut-off of 1e-10)**. Comparison of ESTs having predicted ORFs (FrameDP 1.03 parameters: method used for the first classification: GC3; minimum length of the predicted peptides: 30; e-value cut-off for considering ncbi-blastx hits: 1e-3; reference protein database: Swissprot, 398 181 entries, August 2009) from the two sets revealed enrichment in different activities (BLAST2GO, Fisher's exact test with a FDR correction).

The unigenes were compared to all published ESTs from other Lepidoptera retrieved from NCBI's dbEST (550 623 entries, June 2009) using BLASTN (Figure 2). 3831 sequences (42.4%) gave no similarity to any other ESTs (e-value cut off: 1e-10). The size of these sequences (mean size: 1041.7 bp) is significantly shorter (t-test, t = 354.283, df = 10402, p value < 2.2e-16) than the size of the 5202 sequences matching with other lepidopteran ESTs (mean size = 1134.7 bp). 1448 (37.8%) of the 3831 sequences without EST match had no predicted ORF, which is also significantly higher than the 847 (16.3%) observed in the set of 5202 sequences with an EST match (Chi2, X-squared = 537.7, df = 1, p-value < 2.2e-16) (Figure 2). The assigned 1448 ORFs, which have no match in other EST libraries, correspond to genes that were never isolated from transcriptomic approaches before and likely represent antennal specific transcripts. The 849 ORFs without EST match but with a gene ontology (GO) classification were compared to the 2766 ORFs with at least one EST match and a GO classification (Additional file 5). This comparison should reflect gene enrichment in the antennal transcriptome. Interestingly, the set showed enrichment in odorant binding, olfactory receptor activity, sensory perception of smell and G-protein coupled receptor signalling pathway, in correlation with the sensory function of this organ.

### Gene identification and functional annotation

**Figure 3:**
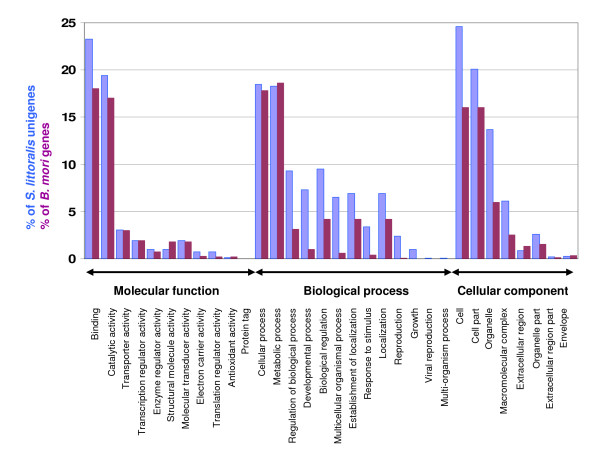
**Distribution of *S. littoralis *unigenes annotated at GO level 2 and comparison with annotated *B. mori *unigene distribution**. The Y-axis shows the percentage of the sequences. The X-axis shows three areas of annotation, and in each area the sequences are further divided into subgroups at GO level 2. *B. mori *GO terms were retrieved from http://www.silkdb.org/cgi-bin/silkgo/index.pl

Figure 3 illustrates the distribution of the *S. littoralis *unigene set in GO terms, compared to the distribution of all *B. mori *genes having GO terms (retrieved from http://www.silkdb.org/cgi-bin/silkgo/index.pl). Among the 6738 *S. littoralis *ORFs, 3619 corresponded to at least one GO term. 3072 were assigned to a molecular function (45.6%), 2586 to putative biological processes (38.4%), and 2282 to a cellular component (33.9%). In the molecular function category, binding and catalytic activities were the most abundant and enriched compared to the *B. mori *genome, in correlation with the results obtained through the specific analyses using available ESTs (see previous paragraph). In the biological process terms, cellular and metabolic processes were the most represented, the other terms being more abundant than in the *B. mori *genome. In the cellular component terms, cell, cell part and organelle were the most abundant and over represented compared to the *B. mori *genome.

### Identification of putative enzymes and secretory proteins

Enzymes are supposed to be abundantly expressed in antennae, as part of the signal termination pathway, but may also participate in neuron protection by xenobiotic degradation [38]. A total of 941 *S. littoralis *ORFs were classified in enzymatic categories using BLAST2GO. In addition to this large analysis, we searched the *S. littoralis *ORF BLASTP results for enzymes expressed in the antennae, with specific key-words corresponding to putative ODEs: carboxylesterase (CXE), glutathione S-transferase and cytochrome P450, which led to the list of 71 unigenes reported in table 2. We found 18 ORFs presenting significant similarities with CXEs. Among the insect putative ODEs, the CXE family is the most studied, and esterase activities were identified in several species that use acetates in their sex pheromone blends [38-42]. In a previous search for CXEs expressed in the antennae of *S. littoralis *(a species that mainly uses acetates as pheromone components) we were able to identify 19 putative esterases [43], among which two were specifically expressed in the antennae. Further comparison of these two sets of esterases will complete the putative CXE repertoire in *S. littoralis *antennae. Other enzyme families proposed to participate in olfactory signal turnover include glutathione S-transferases and cytochrome P450, which can modify odorants to produce odour-inactive compounds [7]. We found 14 and 39 ORFs presenting high similarities with glutathione S-transferases and cytochrome P450, respectively. Our analysis confirms that antennae are a hot-spot for enzymatic activities, as suggested by the previous analysis of antennal ESTs from *E. postvitana *[34]. The role of all these enzymes in olfaction remains to be studied, since these enzymes could also be involved in other processes, such as xenobiotic degradation [38].

**Table 2:** Accession numbers of *S. littoralis *unigenes annotated as encoding Carboxylesterases (CXEs), Glutathione-S-transferases (GSTs), Cytochrome P450 (CYPs) and related enzymes.

CXEs	GSTs	CYPs	NADPH CYP reductases
EZ981801	FQ023109	FQ024248	EZ980988
FQ022303	FQ020799	EZ981137	FQ017461
EZ982197	FQ022743	FQ021196	
FQ019114	FQ019149	FQ021671	
EZ983083	GW825667	FQ019544	
FQ022545	FQ017387	HO118356	
EZ983369	EZ982764	EZ982595	
FQ023061	HO118396	EZ981458	
FQ015927	FQ027411	EZ981571	
FQ022747	FQ018875	FQ018915	
FQ020250	GW826417	EZ981789	
EZ982323	FQ019239	EZ981875	
EZ982624	EZ981154	EZ983482	
EZ982921	EZ982089	FQ015142	
EZ983638		FQ014678	
EZ983598		GW825436	
FQ022223		FQ022752	
FQ020727		FQ028570	
		FQ015778	
		EZ982503	
		FQ020310	
		FQ018184	
		EZ981487	
		FQ018799	
		EZ982387	
		EZ982392	
		EZ983317	
		EZ983029	
		EZ982194	
		EZ982440	
		EZ981393	
		EZ982843	
		EZ981535	
		EZ983216	
		FQ028959	
		EZ982050	
		EZ983461	

The olfactory process within the antennae is thought to be triggered by a large family of proteins, the so-called OBPs, secreted in the sensillum lymph [8]. We thus found it relevant to search for secretory proteins through SignalP [44] in all the translated *S. littoralis *ORFs. A total of 636 translated ORFs (84.1%) were predicted to contain a signal peptide, among which 565 (88.8%) have matches with known proteins in the NR protein database. Among them, candidate binding proteins were found. A complete analysis of candidate OBPs found in the *S. littoralis *ESTs is detailed in the paragraph below. The remaining 11.2% of the putative *S. littoralis *secretory proteins did not share significant similarity with known proteins.

### Identification of putative *S. littoralis *odorant-binding proteins

35 putative *S. littoralis *OBP (SlitOBP) and 12 putative CSP (SlitCSP) fragments were first extracted from the unigenes by scanning the Interproscan result for the Interpro accession IPR006170, TBLASTN search in NR, and specific TBLASTN search among the ESTs with the *B. mori *OBP [45] and CSP [46] (further defined as BmorOBP and BmorCSP) complete repertoires as queries. After a detailed analysis of sequence alignments, several contigs and/or singletons that were not automatically assembled were considered to encode a same putative protein (Table 3). In some cases, pairwise alignments revealed a few nucleotide mismatches, which possibly mirror polymorphism or enzyme errors in the cDNA synthesis process (*eg *EZ982609/FQ016892). In other cases, alignments revealed the presence of large inserts within nucleotide sequences, likely corresponding to unspliced introns. GT splice donor sites were found at the beginning of these inserts and their location appeared to be similar to intron locations described in *B. mori *OBP and CSP genes [45,46] (*eg *EZ981038/EZ982027 for OBPs; EZ983373/GW825922 for CSPs). In total, we annotated 17 SlitOBPs (including six ABPs) and nine SlitCSPs (including one SAP) listed in table 3. Some SlitOBP/CSP unigenes were incomplete at their 5' ends and the corresponding proteins missed the signal peptide (Table 3).

**Table 3:** List of *S. littoralis *unigenes putatively involved in olfactory binding

	Unigene reference	EST nb	Frame	ORF size (aa)	Blastx best hit Reference/Sequence name/species	E value	Identities	Signal peptide
**Pheromone-binding proteins**								
	EZ981038 (EZ982027)	2	+3	102	gb|AAZ22339.1|pheromone binding protein 2 [Spodoptera litura]	3e-41	99%	YES
	EZ983456 (GW825701)	2	+1	163	gb|AAO16091.1|pheromone binding protein 3 [Helicoverpa armigera]	3e-76	83%	YES
	EZ982949 (FQ021292)	95	-3	164	gb|ABQ84981.1|pheromone-binding protein 1 [Spodoptera littoralis]	1e-91	100%	YES
**General odorant-binding proteins**								
	EZ981811 (EZ982114)	8	+2	151	gb|ABM54824.1|general odorant-binding protein GOBP2 [Spodoptera litura]	2e-84	96%	YES
	EZ982647 (FQ018693)	8	-1	163	gb|ABM54823.1|general odorant-binding protein GOBP1 [Spodoptera litura]	1e-81	97%	YES
**Other odorant-binding proteins**								
	EZ983297 (EZ982153, FQ030234, FQ024265, FQ029312)	9	+3	147	emb|CAC33574.1|antennal binding protein [Heliothis virescens]	6e-54	72%	YES
	EZ983149	2	+3	142	gb|AAL60416.1|AF393491_ 1antennal binding protein 2 [Manduca sexta]	5e-53	65%	YES
	EZ981335 (FQ017058)	140	-1	137	emb|CAA05508.1|antennal binding protein X [Heliothis virescens]	4e-53	92%	YES
	FQ026764 (FQ031480, HO118380)	1	+3	141	gb|AAL60413.1|AF393488_ 1antennal binding protein 3 [Manduca sexta]	1e-52	74%	YES
	FQ019778	1	+2	129	gb|AAL60425.1|AF393500_ 1antennal binding protein 7 [Manduca sexta]	1e-30	57%	YES
	EZ981091 (FQ019778, FQ018991)	2	+1	147	gb|AAX98168.1|pheromone binding protein 4 [Spodoptera frugiperda]	1e-72	92%	YES
	FQ020630	1	+3	174	gb|AAO41344.1|Odorant-binding protein 59a [Drosophila melanogaster]	0.27	27%	NO
	FQ021259 (FQ018920, FQ015705)	1	+2	184	emb|CAR85645.1|odorant-binding protein 4 [Myzus persicae]	2e-34	44%	YES
	FQ021928	1	+1	62	ref|NP_ 001140187.1|odorant-binding protein 3 [Bombyx mori]	3e-10	53%	NO
	FQ021918	1	+3	75	ref|NP_ 001140191.1|odorant-binding protein 7 [Bombyx mori]	1e-18	64%	NO
	FQ014244	1	+3	239	dbj|BAH79158.1|odorant binding protein [Bombyx mori]	2e-58	49%	YES
	EZ983259 (FQ026546, FQ020478)	31	+3	252	dbj|BAH79159.1|odorant binding protein [Bombyx mori]	1e-93	60%	YES
**ChemoSensory Proteins**								
	FQ024845 (FQ020519)	1	+2	113	gb|AAK14793.1|sensory appendage protein-like protein [Mamestra brassicae]	1e-30	67%	YES
	EZ981604 (FQ018666)	6	-3	128	gb|AAN63675.1|AF448448_ 1chemosensory protein [Helicoverpa zea]	2e-48	81%	YES
	EZ983373 (GW825922 EZ982648 HO118345)	3	-2	120	ref|NP_ 001037180.1|chemosensory protein CSP2 [Bombyx mori]	4e-45	64%	YES
	EZ982930	4	+1	148	gb|ABM67686.1|chemosensory protein CSP1 [Plutella xylostella]	6e-40	54%	YES
	EZ982103	11	-3	266	ref|NP_ 001037069.1|chemosensory protein 8 [Bombyx mori]	5e-58	86%	YES
	EZ982609 (FQ016892)	4	+1	127	gb|AAV34687.1|chemosensory protein 2 [Heliothis virescens]	1e-48	87%	YES
	EZ983355	3	+1	123	ref|NP_ 001037065.1|chemosensory protein 6 [Bombyx mori]	6e-41	60%	YES
	GW825956	1	+1	128	gb|ABM67689.1|chemosensory protein CSP2 [Spodoptera exigua]	9e-59	96%	YES
	HO118383 (FQ020895)	1	+2	122	ref|NP_ 001091779.1|chemosensory protein 11 [Bombyx mori]	3e-29	80%	YES

Interestingly, using Interproscan, a new putative OBP type was found (FQ020630) that presented no sequence identity with any known insect OBPs (only 27% identity with its BLASTX best hit *D. melanogaster *OBP59a, e-value 0.27, unexpected for insect OBPs). The deduced encoded protein seemed to be complete (with start and stop codons) but SignalP analyses did not reveal the occurrence of a signal peptide, suggesting that this protein is not secreted or that we missed its N-terminal part. This latter hypothesis is supported by the fact that the amino acid sequence contains only five cysteine residues, one less than usually observed in insect OBPs. Alternatively, this sequence could encode a protein belonging to the Takeout or juvenile hormone-binding protein families, since these protein families also bind lipophilic molecules. However, alignment with Lepidoptera Takeout or juvenile hormone-binding proteins was not possible. Further molecular (*eg *localisation of the expressing cells) and functional (*eg *binding properties) analyses are now needed to definitely annotate this new protein as an OBP.

Although the best BLASTX hit for two unigenes, the contig EZ983259 and the singleton FQ014244, consisted of putative BmorOBPs, they could not be aligned with insect OBPs and both presented similarity with juvenile hormone-binding protein and Takeout-like proteins. Thus, they were not included in the following phylogenetic analyses.

**Figure 4:**
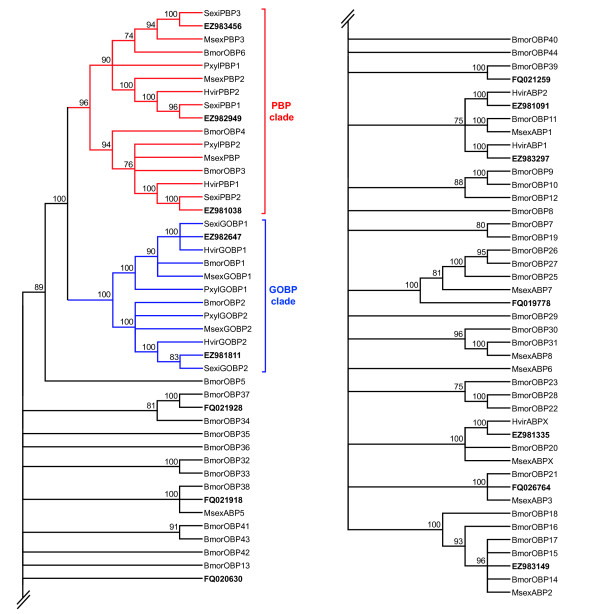
**Phylogenetic tree of candidate odorant-binding proteins from *S. littoralis*, *B. mori *and other lepidopterans, including pheromone-binding protein (PBP) and general odorant-binding protein (GOBP) clades**. The *S. littoralis *translated unigenes are shown in bold. Accession numbers are given in additional file 6. The tree was constructed with MEGA4, using the neighbour-joining method. Values indicated at the nodes are boostrap percentages based on 1000 replicates, and nodes with bootstrap values < 70% were collapsed. Bmor, *Bombyx mori*; Hvir, *Heliothis virescens*; Msex, *Manduca sexta*; Pxyl, *Plutella xylostella*; Sexi, *Spodoptera exigua*.

A phylogenetic analysis of OBPs (Figure 4) was carried out using protein sequences from *S. littoralis*, *B. mori *and other Lepidoptera (accession numbers provided in additional file 6). In view of these analyses, at least one lepidopteran orthologue could be found for each putative SlitOBP identified, except for the new type of OBP identified in this study (FQ020630). In particular, we were able to annotate three SlitPBPs (EZ982949 = SlitPBP1; EZ981038 = SlitPBP2; EZ983456 = SlitPBP3) and two SlitGOBPs (EZ982647 = SlitGOBP1 and EZ981811 = SlitGOBP2). Since we found one candidate in each of the three lepidopteran PBP lineages and in each of the two GOBP lineages, it suggests that we identified the complete repertoire of these OBP families in *S. littoralis*. Interestingly, the BLASTX best hit for EZ981091 was *S. frugiperda *PBP4, but the phylogenetic position of the protein translated from this contig strongly argues that it does not belong to the PBP family.

### Identification of *S. littoralis *candidate chemosensory receptors

#### 31 candidate chemosensory receptors were identified in male antennae

The chemosensory receptor family includes ORs and gustatory receptors (GRs). In *B. mori*, the recently available sequenced genome offered the opportunity to identify the almost complete repertoires of ORs [20,21] and GRs [47] in a lepidopteran species. 41 candidate *B. mori *ORs (BmorORs) previously identified [20] were compared to the unigenes using TBLASTN, leading to a first identification of 25 putative ORs in *S. littoralis *(SlitORs). Only six BmorORs gave no result when used as query sequences (BmorOR17, 18, 21, 22, 23 and 43). In parallel, the Interproscan result was scanned to retrieve sequences including one or more domains related to olfactory reception (IPR004117), resulting in the identification of seven putative ORs. Among them, three new sequences (EZ982994, EZ981047, FQ015038) were not identified during the precedent analysis using BmorORs. Additional searches using described insect OR families (*D. melanogaster, A. gambiae, A. mellifera, T. castaneum, Aedes aegypti*), as well as some isolated lepidopteran sequences [22-24,26,27] and additional BmorORs recently identified [21] led to the identification of a total of 35 candidate chemosensory receptor partial sequences: 33 olfactory receptors (SlitORs) and two gustatory receptors (SlitGRs). These *S. littoralis *sequences were in turn employed in searches to find more genes in an iterative process, which did not lead to the identification of additional candidates.

As for OBPs and CSPs, some ORFs appeared to overlap with a high sequence identity, and sequence alignments were further manually analyzed. We propose that the following unigenes encode a single protein: EZ982777/FQ025462 (residual intron in the latter, identified by the presence of intron/exon boundaries), EZ981960/FQ025873/FQ021134 (incomplete 5' end for the singletons and presence of several punctual mutations) and FQ023155/FQ021957. The final number of candidate chemosensory receptors identified is then 31, including 29 ORs and two GRs, and the corresponding unigenes are listed in table 4.

**Table 4:** List of *S. littoralis *unigenes putatively involved in chemosensory reception

	Unigene reference	EST nb	Frame	ORF size (aa)	Blastx best hit Reference/Sequence name/species	E value	Identity	Antennae enriched
**Candidate olfactory receptors**								
	EZ982621	3	+1	307	ref|NP_ 001103623.1|odorant receptor 33 [Bombyx mori]	2e-43	31%	YES
	EZ981394	5	-2	291	ref|NP_ 001091790.1|candidate olfactory receptor [Bombyx mori]	8e-98	65%	YES
	EZ981024	2	3	335	dbj|BAH66326.1|olfactory receptor [Bombyx mori]	1e-127	70%	YES
	EZ981047	11	-1	302	gb|ABQ82137.1|chemosensory receptor 2 [Spodoptera littoralis]	8e-147	88%	YES
	EZ983328	7	+3	285	emb|CAG38117.1|putative chemosensory receptor 16 [Heliothis virescens]	1e-88	57%	YES
	EZ981960 (FQ025873, FQ021134)	6	-2	263	emb|CAG38117.1|putative chemosensory receptor 16 [Heliothis virescens]	5e-99	65%	YES
	EZ983448	5	-1	329	emb|CAD31853.1|putative chemosensory receptor 7 [Heliothis virescens]	2e-08	26%	YES
	EZ982994	5	1	299	ref|NP_ 001155301.1|olfactory receptor BmOr-60 [Bombyx mori]	2e-106	72%	YES
	EZ982443	4	-1	274	dbj|BAH66361.1|olfactory receptor [Bombyx mori]	5e-13	45%	N/A
	EZ981646	4	-3	264	ref|NP_ 001103476.1|odorant receptor 35 [Bombyx mori]	1e-76	55%	NO
	EZ982777 (FQ025462)	3	+1	299	dbj|BAG71423.2|olfactory receptor [Mythimna separata]	8e-106	57%	NO
	EZ981417	3	+2	282	dbj|BAH66359.1|olfactory receptor [Bombyx mori]	8e-30	70%	YES
	EZ983476	2	+1	331	emb|CAG38119.1|putative chemosensory receptor 18 [Heliothis virescens]	1e-137	73%	YES
	EZ983129	2	-2	301	dbj|BAH66312.1|olfactory receptor [Bombyx mori]	4e-69	42%	YES
	EZ983645	2	+1	165	emb|CAG38122.1|putative chemosensory receptor 21 [Heliothis virescens]	1e-67	58%	NO
	EZ982362	2	-1	173	ref|NP_ 001104830.1|odorant receptor 41 [Bombyx mori]	0.95	19%	NO
	EZ981187	2	+1	199	gb|ACJ12929.1|odorant receptor 3 [Epiphyas postvittana]	1e-95	63%	YES
	EZ982965	2	+2	250	tpg|DAA05977.1|TPA: TPA_ exp: odorant receptor 19 [Bombyx mori]	1e-40	39%	YES
	GW825563		+1	250	dbj|BAH66339.1|olfactory receptor [Bombyx mori]	2e-65	82%	YES
	FQ023155 (FQ021957)		+3	189	emb|CAG38118.1|putative chemosensory receptor 17 [Heliothis virescens]	5e-74	76%	N/A
	FQ031836		+3	239	ref|NP_ 001104797.1|odorant receptor 30 [Bombyx mori]	1e-41	43%	YES
	FQ031000		+3	223	gb|ACS45305.1|candidate odorant receptor 11 [Helicoverpa armigera]	2e-109	85%	YES
	FQ030158		+1	254	emb|CAD31949.1|putative chemosensory receptor 8 [Heliothis virescens]	4e-49	50%	YES
	FQ029014		+3	217	gb|ACM18061.1|putative odorant receptor OR3 [Manduca sexta]	1e-09	28%	N/A
	FQ016760		+2	266	ref|XP_ 312381.1|candidate odorant receptor (AGAP002558-PA) [Anopheles gambiae str. PEST	23	28%	YES
	FQ017398		+2	310	ref|NP_ 001091789.1|candidate olfactory receptor [Bombyx mori]	2e-57	49%	YES
	FQ018861		+2	229	dbj|BAH66348.1|olfactory receptor [Bombyx mori]	2e-44	35%	N/A
	FQ014255		+2	258	ref|NP_ 001103476.1|odorant receptor 35 [Bombyx mori]	1e-73	48%	YES
	FQ015038		+3	285	ref|NP_ 001116817.1|olfactory receptor-like receptor [Bombyx mori]	1e-94	60%	YES
**Candidate gustatory receptors**								
	FQ016677		+2	267	ref|XP_ 001848689.1|gustatory receptor 24 [Culex quinquefasciatus]	3e-41	38%	NO
	GW825869		+3	227	ref|XP_ 001654839.1|Gustatory receptor 21a, putative [Aedes aegypti]	4e-73	57%	N/A

#### Putative gustatory receptors expressed in adult antennae

The two GR candidates are, to our knowledge, the first identified in Lepidoptera antennae. It is not surprising to find candidate GRs since these organs are known to carry some taste sensilla [48]. Interestingly, one of these GRs (GW825869) presented similarity with members of the GR21a family (Table 4). In *Drosophila*, GR21a forms a heteromeric receptor in combination with GR63a, which allows the detection of CO_2 _[49,50]. Putative CO_2 _receptors have been described in *B. mori *[47], one of which (BmGr2NJ as described by [47]) presented 78% identity with the partial sequence we obtained in *S. littoralis*. Since CO_2 _receptors are quite conserved among insects [51], this high sequence identity supports the annotation of this *S. littoralis *GR as a candidate CO_2 _receptor. Receptor cells to CO_2 _were found on the antennae of some insect species, such as the honey bee [52] and *Drosophila *[53], but up to now, moth receptor cells for CO_2 _have been only described on labial palps [54]. Thus, annotation of this GR as a candidate CO_2 _receptor awaits further demonstration of CO_2 _detection by *S. littoralis *antennae.

#### Annotation of the S. littoralis putative ORs

In *S. littoralis*, 63 glomeruli could be identified in the antennal lobe [55]. Considering the one receptor-one glomerulus paradigm [56,57], by which the number of expected ORs in a given species should correlates with the number of glomeruli in the antennal lobe, we estimate that the 29 candidate OR genes identified represent half of the *S. littoralis *OR repertoire. 45% of the SlitOR amino acid sequences showed low sequence conservation with already known receptor proteins (less than 50% identity with the best hit, table 4). The remaining SlitORs presented higher conservation (up to 88% identity with the best hit), and eight SlitORs (26%) shared more than 70% identity with their respective best hits (Table 4). Among these conserved sequences, we could recognize SlitOR2 (the *D. melanogaster *OR83b orthologue, translated from EZ981047) and SlitOR18 (EZ983476), two *S. littoralis *receptors that we previously identified by homology cloning [25,31]. The predicted translations of four unigenes exhibited a high conservation level (from 57 to 85% identity, table 4) with lepidopteran PRs previously described [21,22,26]. Since lepidopteran PRs form a relatively well conserved lineages [24], these unigenes could encode candidate PRs in *S. littoralis*.

**Figure 5:**
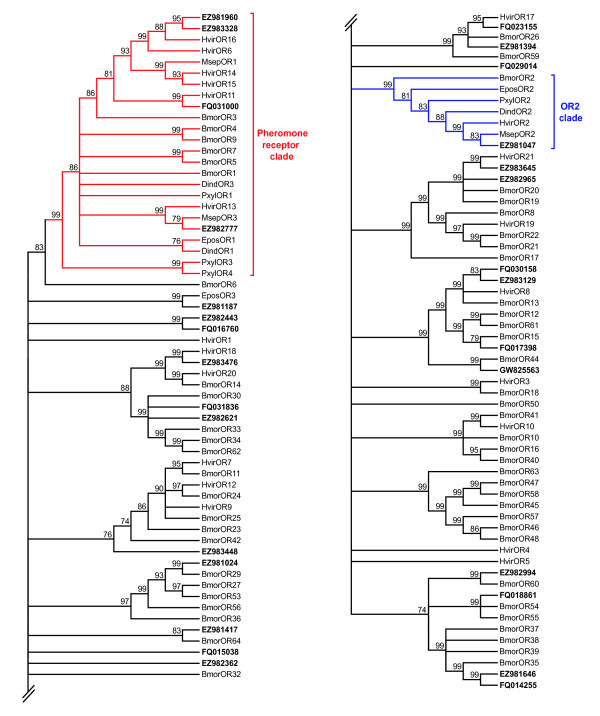
**Phylogenetic tree of candidate olfactory receptors (ORs) from *S. littoralis*, *B. mori *and other lepidopterans, including pheromone receptor (PR) and OR2 (*D. melanogaster *OR83b orthologue) clades**. The *S. littoralis *translated unigenes are shown in bold. Accession numbers are given in additional file 6. The tree was constructed as in figure 4. Bmor, *Bombyx mori*; Dind, *Diaphania indica*; Epos, *Epiphyas postvittana*; Hvir, *Heliothis virescens*; Msep, *Mythimna separata*; Pxyl, *Plutella xylostella*.

A phylogenetic analysis was conducted with the putative SlitORs and other lepidopteran OR sequences, including the annotated BmorORs (accession numbers provided in additional file 6) (Figure 5). At least one lepidopteran orthologue could be assigned to the majority of the putative SlitORs, only five of them having no counterpart. Without surprise, the highly conserved SlitOR2 (EZ981047) clustered with other OR2 sequences (*D. melanogaster *OR83b orthologues) (Figure 5). In correlation with the BLAST results, the four candidate SlitPRs clustered in the lepidopteran PR clade, supporting their annotation.

#### qPCR analysis of S. littoralis chemosensory receptors

**Figure 6:**
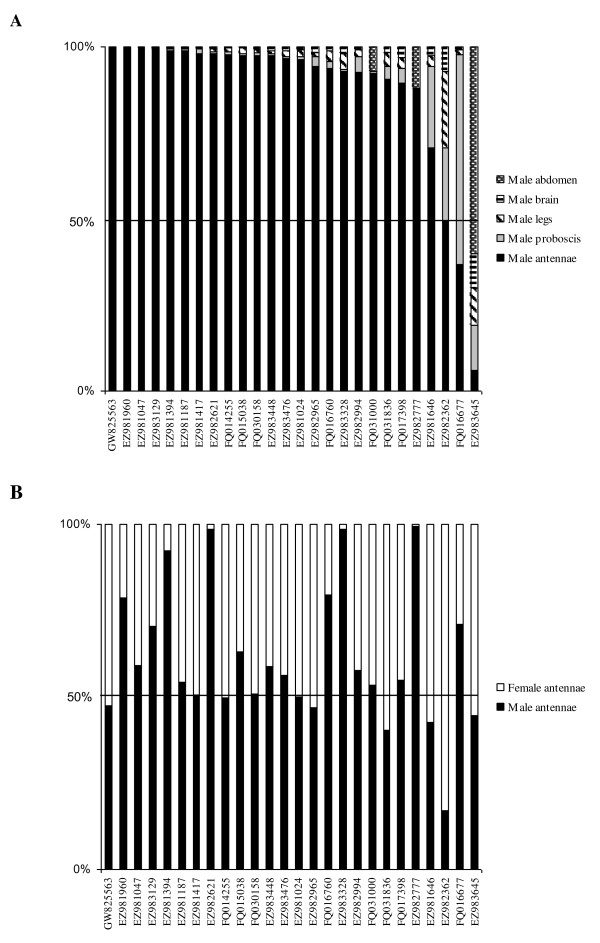
**Distribution pattern of *S. littoralis *candidate olfactory and gustatory receptors in different male tissues (A) and in male and female antennae (B)**. Gene expression levels were determined by real-time PCR and calculated relatively to the expression of the rpL8 control gene and expressed as the ratio = E_SlitCR_^(ΔCT ^^SlitCR)^/E_rpL8 _^(ΔCT rpL8)^. The Y-axis shows the percentage of expression in each tissue with a total of 100% and the X-axis reports the accession numbers of the candidate olfactory and gustatory receptors. See table 4 for unigene details.

Insect chemosensory receptors usually exhibit a specific or enriched expression in chemosensory organs [13,20,27]. We thus conducted a preliminary study on a single set of samples and considering a single time point, using quantitative real-time PCR, to address the tissue-distribution of the candidate chemosensory receptors we identified in *S. littoralis*. Data were obtained for 26 unigenes (Figure 6A). For the others, we encountered primer design problems and/or bad efficiencies. As expected, most of our candidates were expressed in a tissue-specific manner, being enriched in chemosensory tissues (antennae and/or proboscis), thus supporting our annotation. EZ983645 was expressed in all tissues tested, unexpected for a chemosensory receptor. One of the two GR candidates expressed in the antennae (FQ016677) appeared to be also well expressed in the proboscis, supporting its annotation. Interestingly, two candidate ORs were well expressed in the proboscis (EZ981646 and EZ982362). Consistent with this observation, a previous study demonstrated that the taste organ of the mosquito *A. gambiae *does express ORs and exhibited olfactory responses [58].

Since pheromone receptors are usually male-specific or male-enriched [22-24,59,60], we next compared SlitOR expression levels between male and female antennae (Figure 6B). Although this preliminary analysis is of limited value, we found that four unigenes were enriched in male antennae (EZ981394, EZ982621, EZ983328 and EZ982777) (Figure 6B). Only two of them (EZ983328 and EZ982777) corresponded to the SlitORs annotated as putative PRs after the BLAST and phylogenetic analyses (see paragraph above). The two additional putative ORs enriched in male antennae (EZ981394 and EZ982621) did not present high sequence identities with other moth PR candidates or functionally characterized PRs.

### Other genes putatively involved in the olfactory process and its modulation

#### Ionotropic receptors (IRs), sensory neuron membrane proteins (SNMPs) and transduction

Recently, the ionotropic receptors (IRs), that constitute a family of ionotropic glutamate receptor-related proteins, have been identified as defining a new class of chemosensory receptors in *D. melanogaster *[61]. The 61 described *D. melanogaster *IRs were used to search for homologues in the *S. littoralis *ESTs by TBLASTN. This led to the identification of five putative *S. littoralis *IRs. However, further studies are needed to annotate these candidates as IRs or classical glutamate receptors, such as obtaining the full length sequences for detailed examination of the binding site.

We also identified two unigenes encoding putative SNMPs, annotated as SNMP1 and SNMP2 in accordance with their best hit (accession numbers: EZ982816 and EZ982501, additional files 1 and 2). SNMPs were first identified in pheromone-sensitive neurons of Lepidoptera [10,62] and are thought to play a role in pheromone detection, as demonstrated for the *D. melanogaster *SNMP1 homologue [11].

Our EST analyses (see above) revealed that the antennae appeared to be enriched in genes involved in metabotropic activity. Among examples, we annotated unigenes putatively encoding proteins such as G-proteins and G-protein related elements, second messenger-related enzymes and ions channels such as voltage-gated ion channels, calcium and chloride channels (additional files 1 and 2). Some of these genes have been previously described in detail, such as a diacylglycerol kinase [63] and a transient receptor potential channel [64], whose function in pheromone signal transduction was suspected. However, the way insect ORs transduce the signal is currently under debate and although a classical metabotropic pathway via G-protein was assumed [65], recent studies proposed an alternative or complementary ionotropic process [66,67].

#### Modulation/regulatory process

Unigenes were identified as encoding proteins putatively involved in modulation/regulatory process, such as hormone receptors (including ecdysone receptors EcR and USP), juvenile hormone-binding proteins, Takeout-like proteins and biogenic amine receptors (additional files 1 and 2). Consistent with the present data, we have previously characterized an octopamine/tyramine receptor expressed in the olfactory sensilla of an other noctuid, *Mamestra brassicae *[68]. Biogenic amines act as neurohormones, neuromodulators or neurotransmitters in most invertebrate species [69], and evidence has been accumulated over the last decades that such biogenic amines participate in the modulation of olfactory reception [70,71]. Ecdysone and juvenile hormone are key hormones involved in the maturation [72] and the plasticity [73] of the olfactory system.

Among our unigenes, we have also annotated putative circadian clock components. In addition to the previously described *period *and *cryptochrome *genes [74], we identified in *S. littoralis *antennae other fragments homolog to circadian clock encoding genes, such as *timeless *and *vrille *(additional files 1 and 2). These data support our previous finding that *S. littoralis *antennae house a peripheral circadian clock [74].

### LepidoDB implementation

Lepido-DB (http://www.inra.fr/lepidodb) is a centralized bioinformatic resource for the genomics of major lepidopteran pests [75]. This Information System was designed to store, organize, display and distribute various genomic data and annotations. Beside a BLAST search and a full text search facilities, the system was constructed using open source software tools from the Generic Model Organism Database (GMOD) including a Chado database. All the data, unigenes, ORFs and their annotation generated in this project have been included in LepidoDB. As a result, from the project page http://www.inra.fr/lepidodb/spodoptera_littoralis one can retrieve the whole sequence set, query with a keyword and retrieve the corresponding sequences.

## Conclusions

The main objective of this study was to identify genes potentially involved in olfactory signal detection in a crop pest model, *S. littoralis*. We annotated a total of ~130 unigenes encoding putative proteins involved in all the steps of ligand detection (transport, docking, recognition, degradation). In particular, the normalization process, alongside with the high number of sequenced ESTs compared to previous antennal libraries, allowed enriching the EST collection in rare or low abundant transcripts. This strategy appeared to be particularly relevant for the identification of new insect chemosensory receptors in a species for which no genomic data are available. Concerning the pheromone detection process, we identified in this species three PBPs, two SNMPs, candidates PRs and many CXEs as putative PDEs, as a prerequisite to further identify which PBP/PR/SNMP/PDE act in concert to ensure the specificity of the recognition process within a given functional type of pheromone-sensitive sensilla. Their respective expression patterns remain to be elucidated to crack the code of their combinatorial expression.

Our analyses also suggest that the olfactory sensitivity may be modulated as early as the antennal level, before signal integration in the brain. Indeed, we annotated a long list of biogenic amine/hormone targets and circadian elements expressed in the antennae, as a first step toward understanding olfactory plasticity at the peripheral level.

Moreover, our study revealed that antennae express abundant defense-related elements involved in xenobiotic and pathogen protection. This observation could be explained by the fact that antennae, whose morphology is adapted to let odorant molecules enter the organism, represent an open space for harmful molecules.

Besides olfaction, insect antennae are involved in different non-olfactory processes, such as taste, balance/gravity, wind and sound sensing [76-78], and, as recently demonstrated, sun compass orientation [79]. The availability of an antennal transcriptome is thus a valuable resource for olfaction and pheromone detection studies, but also for investigation of the molecular bases of other antennal functions.

## Methods

### Insect rearing and male antennae cDNA library construction

Insects originated from our inbred laboratory strain of *S. littoralis*. Insects were reared on semi-artificial diet [80], under 23°C, 60-70% relative humidity and 16:8 light:dark cycle. Pupae were sexed and males and females were kept separately. Antennae were collected from 1-2 day old naïve adult males and stored at -80°C until we obtained a total number of 12 000 antennae. Two mg of total RNA were isolated using the TriZol reagent (Invitrogen, Carlsbad, CA, USA), quantified in a spectrophotometer, and the quality verified by agarose gel electrophoresis. A custom normalized EvoQuest™ cDNA library was created by Invitrogen in the pSPORT 6.1 vector, using 1 mg of total RNA as starting material, without any amplification. The normalization step (performed by Invitrogen) introduced in the library construction consisted of a single stranded antisense DNA target-biotinylated sense RNA driver hybridization and a capture with streptavidin to remove target/driver hybrids and un-hybridized drivers, leftover DNA representing the normalized library. This led to a 20 fold reduction of abundant genes as measured for β-actin, while maintaining a good average insert size of 2.1 kb. The normalization procedure was used to minimize EST redundancy and to enrich the library for rare and low abundant genes, to allow new gene discovery.

### EST sequencing

The library was plated, and 2400 clones were randomly picked. Their 5' ends were sequenced using REV primer (Genome-express, Grenoble, France). 93% of the clones (2218) presented an insert, we thus undertook a high-throughput sequencing project (20000 sequences) in partnership with the Genoscope (Evry, France). The plated library was arrayed robotically and bacterial clones had their plasmid DNA amplified using phi29 polymerase. The plasmids were end-sequenced using BigDye Termination kits on Applied Biosystems 3730xl DNA Analysers. Adaptor and vector were localized using cross_match (http://www.phrap.org/) using default matrix (1 for a match, -2 penalty for a mismatch), with mean scores of 6 and 10, respectively. Sequences were then trimmed following three criteria: vector and adaptor, poly(A) tail or low quality (defined as at least 15 among 20 bp with a phred score below 12). Moreover, while submitting the unigenes to GenBank, the sequences were also compared to known vectors using the vecscreen software (http://www.ncbi.nlm.nih.gov/VecScreen/VecScreen.html). We finally obtained the 5' end sequences of 20760 ESTs.

### Sequence processing, assembly, unigene and peptide generation

EST assembly was processed using the TGI Clustering tools (TGICL, http://compbio.dfci.harvard.edu/tgi/software) using the default parameters (minimum percent identity for overlaps: 94, minimum overlap length: 30 bp, maximum length of unmatched overhangs: 30), generating unigenes and singletons. Peptides were extracted from the unigenes using FrameDP 1.03 [81], with three training iterations and using Swissprot (398 181 entries, August 2009) as reference protein database.

### Specificity analysis using EST

The unigenes were compared to the 550623 lepidopteran ESTs retrieved from the NCBI Entrez server (July 2009), using BLASTN with an e-value cut-off of 1e-10. The analysis of the enrichment of the EST library was performed with the help of the BLAST2GO application [82] using GOSSIP [83]. In this application, GO terms are tested for enrichment in a test group when compared to a reference group using Fisher's exact test with multiple testing correction. The statistical tests were achieved with the R t-test and Chi2 methods.

### Gene identification and functional annotation

The unigenes were compared to the NCBI non redondant protein database (7686184 entries, July 2009 version), the FlyBase translational database v5.16 [84] (21 647 entries) and 14623 *B. mori *Glean peptide predictions from SilkDB (April 2008 release, ftp://silkdb.org/pub/current/Gene/Glean_genes/silkworm_glean_pep.fa.tar.gz) [85] using BLASTX, with a 1e-5 e-value threshold. The Gene Ontology mapping and distribution were done with the help of BLAST2GO (GO association done by a BLAST against the NCBI NR database). Finally, the functional domain protein profile and domain were predicted by queries against InterPro using InterproScan [37], running a batch of analyses (BLASTProDom, Coil, FprintScan, Gene3D, HMMPanther, HMMPfam, HMMPIR, HMMSmart, HMMTigr, PatternScan, ProfileScan, RNA-BINDING, Seg and Superfamily) on the predicted ORFs. The protein sequences were searched for the occurrence of a signal peptide using SignalP 3.0 [44].

### Identification of odorant-binding proteins and chemosensory receptors

The *S. littoralis *antennal unigenes were searched with *B. mori *OBPs, CSPs, chemosensory receptors and all available insect ORs retrieved from Swissprot as queries using TBLASTN [86]. Additionally, the Interproscan results were scanned for the Interpro accession IPR006170 (Pheromone/general odorant-binding protein, PBP/GOBP Molecular Function: odorant binding GO:0005549) and IPR004117 (Molecular Function: olfactory receptor activity GO:0004984). *S. littoralis *putative chemosensory receptor sequences were in turn employed in searches to find more genes in an iterative process.

### Phylogenetic analyses

We built OBP and OR neighbor-joining trees based on Lepidoptera data sets. The OBP data set contained the 43 complete amino acid sequences deduced from the genome of *B. mori*, together with the largest OBP repertoires characterized within noctuid moths (7 sequences from *H. virescens *and 5 from *Spodoptera exigua*) and outside noctuid moths (13 from *M. sexta *and 4 from *Plutella xylostella*) (Accession numbers available in additional file 6). Signal peptide sequences were removed following predictions of cleavage site location made by SignalP 3.0. The OR data set contained 58 amino acid sequences from *B. mori *(6 sequences were removed from the alignment because of their short length) and the 21 sequences characterized from *H. virescens*, completed with subsets of sequences characterized within noctuids (3 sequences from *Mythimna separata*) and outside noctuids (4 from *P. xylostella*, 3 from *Diaphania indica *and 3 from *E. postvittana*). Amino acid sequences were aligned using ClustalW2 [87]. Unrooted trees were constructed by the neighbour-joining method, with Poisson correction of distances, as implemented in MEGA4 software [88]. Node support was assessed using a bootstrap procedure base on 1000 replicates, and nodes supported by a bootstrap value under 70% were collapsed to an horizontal line when drawing cladograms.

### Quantitative real-time PCR

Naïve males and females in the middle of their second scotophase were used in the following experiments. Male antennae, proboscis, legs (mixture of front, middle, and hind legs), brains, abdomens and female antennae were collected and stored at -80°C. Total RNAs were extracted with the RNeasy^® ^MicroKit (Qiagen, Hilden, Germany) that included a DNase treatment. Single-stranded cDNAs were synthesized from 1 μg of total RNAs with 200 U of M-MLV reverse transcriptase (Clontech, Mountain View, CA, USA) using buffer and protocol supplied in the Advantage^® ^RT-for-PCR kit (Clontech). Gene-specific primers for *S. littoralis *chemosensory receptors and the endogenous control rpL8 were designed using the Beacon Designer 4.0 software (Bio-Rad, Hercules, CA, USA), yielding PCR products ranging from 100 to 250 bp (see additional file 7). qPCR mix was prepared in a total volume of 20 μl with 10 μl of Absolute QPCR SYBR Green Mix (ThermoFisher Scientific, Epsom, UK), 5 μl of diluted cDNA (or water for the negative control or RNA for controlling for the absence of genomic DNA) and 200 nM of each primer. qPCRs were performed on *S. littoralis *cDNAs using a MJ Opticon Monitor Detection System (Bio-Rad). The PCR program began with a cycle at 95°C for 15 min, followed by 40 cycles of 20 s at 95°C, 15 s at 53 to 62°C (depending on the primer pair) and 20 s at 72°C. To assess the purity of the PCR reactions, a dissociation curve of the amplified product was performed by gradual heating from 50°C to 95°C at 0.2°C/s. Standard curves were generated by a five-fold dilution series of a cDNA pool evaluating primer efficiency E (E = 10^(-1/slope)^). All reactions were performed in duplicate. Chemosensory receptor expression levels were calculated relatively to the expression of the rpL8 control gene and expressed as the ratio = E_SlitCR_^(ΔCT ^^SlitCR)^/E_rpL8 _^(ΔCT rpL8) ^[89].

## List of abbreviations

ABP: antennal-binding protein; CSP: chemosensory protein; CXE: carboxylesterase; EST: expressed sequenced tag; GOBP: general odorant-binding protein; GR: gustatory receptor; OBP: odorant-binding protein; ODE: odorant-degrading enzyme; OR: olfactory receptor; ORF: open reading frame; PBP: pheromone-binding protein; PDE: pheromone-degrading enzyme; PR: pheromone receptor; SNMP: sensory neuron membrane protein

## Authors' contributions

FL, SM and FC carried out the bioinformatic analyses. NM performed the sequence alignments and the phylogenetic analyses. ChMe and MCF collected the antennae and prepared the RNA, and ChMe participated in drafting the manuscript. MMC participated in olfactory gene annotation and in drafting the manuscript. FG and JP coordinated and performed the EST sequencing at Genoscope Evry. ChMo designed and performed the qPCR analyses. EJJ conceived and coordinated the study, participated in olfactory gene annotation and in drafting the manuscript. All authors read and approved the final manuscript.

## Supplementary Material

Additional file 1 **Full description of *S. littoralis *ESTs, including accession number, sequence, length, accession number of the corresponding contig if any and blast hit**. Click here for file

Additional file 2 **Full description of *S. littoralis *unigenes, including accession number, gene size, ORF length, EST coverage, blast, signalP analysis, unigene sequence, ORF sequence**. Click here for file

Additional file 3 **Table of the GO terms over or under represented in *S. littoralis *ORFs having no similarity with the *B. mori *proteome**. Test group: ORFs with no match. Reference group: ORFs with at least one match. Fisher's exact test with multiple testing correction. Click here for file

Additional file 4 **Table of the Interproscan results for the 678 *S. littoralis *unigene sequences having no similarity with any known proteins**. Click here for file

Additional file 5 **Table of the GO terms over or under represented in *S. littoralis *unigenes having no lepidopteran EST match**. Test group: *S. littoralis *ESTs with no match. Reference group: *S. littoralis *ESTs with at least one match. Fisher's exact test with multiple testing correction. Click here for file

Additional file 6 **Accession numbers for amino acid sequences of the lepidopteran odorant-binding proteins, antennal binding proteins, chemosensory proteins and olfactory receptors used in the phylogenetic analyses**. Click here for file

Additional file 7 **Table reporting the forward and reverse primer sequences used in real-time PCR, annealing temperatures, resulting amplicon lengths and PCR efficiencies**. Click here for file
