# Supplementary material for: An Expressed Sequence Tag collection from the male antennae of the Noctuid moth Spodoptera littoralis: a resource for olfactory and pheromone detection research
Source: BMC Genomics. 2011 Jan 29;12:86. doi: 10.1186/1471-2164-12-86 (PMC3045336; doi:10.1186/1471-2164-12-86)
Supplement: Additional file 3 — Table of the GO terms over or under represented in S. littoralis ORFs having no similarity with the B. mori proteome. Test group: ORFs with no match. Reference group: ORFs with at least one match. Fisher's exact test with multiple testing correction. [file 1471-2164-12-86-S3.DOC]

| **GO Term** | **Name** | **FDR** | **FWER** | **single test p-Value** | **# in test group** | **# in reference group** | **# non annot test** | **# non annot reference group** | **Over/Under** |
| --- | --- | --- | --- | --- | --- | --- | --- | --- | --- |
| [GO:0043234](../../../../gossipinfo/GO/0043234) | protein complex | 0 | 0 | 0,00404353 | 1 | 515 | 49 | 3050 | under |
| [GO:0005811](../../../../gossipinfo/GO/0005811) | lipid particle | 0 | 0 | 0,091077 | 0 | 168 | 50 | 3397 | under |
| [GO:0043436](../../../../gossipinfo/GO/0043436) | oxoacid metabolic process | 0 | 0 | 0,132969 | 0 | 142 | 50 | 3423 | under |
| [GO:0042623](../../../../gossipinfo/GO/0042623) | ATPase activity, coupled | 0 | 0 | 0,190812 | 0 | 117 | 50 | 3448 | under |
| [GO:0030529](../../../../gossipinfo/GO/0030529) | ribonucleoprotein complex | 0 | 0 | 0,199229 | 0 | 114 | 50 | 3451 | under |
| [GO:0048610](../../../../gossipinfo/GO/0048610) | reproductive cellular process | 0 | 0 | 0,199229 | 0 | 114 | 50 | 3451 | under |
| [GO:0046483](../../../../gossipinfo/GO/0046483) | heterocycle metabolic process | 0 | 0 | 0,233321 | 0 | 103 | 50 | 3462 | under |
| [GO:0044451](../../../../gossipinfo/GO/0044451) | nucleoplasm part | 0 | 0 | 0,257926 | 0 | 96 | 50 | 3469 | under |
| [GO:0006911](../../../../gossipinfo/GO/0006911) | phagocytosis, engulfment | 0 | 0 | 0,265412 | 0 | 94 | 50 | 3471 | under |
| [GO:0003006](../../../../gossipinfo/GO/0003006) | reproductive developmental process | 0 | 0 | 0,269234 | 0 | 93 | 50 | 3472 | under |
| [GO:0022804](../../../../gossipinfo/GO/0022804) | active transmembrane transporter activity | 0 | 0 | 0,28507 | 0 | 89 | 50 | 3476 | under |
| [GO:0005739](../../../../gossipinfo/GO/0005739) | mitochondrion | 0 | 0 | 0,289169 | 0 | 88 | 50 | 3477 | under |
| [GO:0030234](../../../../gossipinfo/GO/0030234) | enzyme regulator activity | 0 | 0 | 0,306152 | 0 | 84 | 50 | 3481 | under |
| [GO:0006886](../../../../gossipinfo/GO/0006886) | intracellular protein transport | 0 | 0 | 0,310548 | 0 | 83 | 50 | 3482 | under |
| [GO:0005506](../../../../gossipinfo/GO/0005506) | iron ion binding | 0 | 0 | 0,310548 | 0 | 83 | 50 | 3482 | under |
| [GO:0030154](../../../../gossipinfo/GO/0030154) | cell differentiation | 0 | 0 | 0,324111 | 0 | 80 | 50 | 3485 | under |
| [GO:0006461](../../../../gossipinfo/GO/0006461) | protein complex assembly | 0 | 0 | 0,333474 | 0 | 78 | 50 | 3487 | under |
| [GO:0006520](../../../../gossipinfo/GO/0006520) | cellular amino acid metabolic process | 0 | 0 | 0,338254 | 0 | 77 | 50 | 3488 | under |
| [GO:0003002](../../../../gossipinfo/GO/0003002) | regionalization | 0 | 0 | 0,343102 | 0 | 76 | 50 | 3489 | under |
| [GO:0030036](../../../../gossipinfo/GO/0030036) | actin cytoskeleton organization | 0 | 0 | 0,343102 | 0 | 76 | 50 | 3489 | under |
| [GO:0044255](../../../../gossipinfo/GO/0044255) | cellular lipid metabolic process | 0 | 0 | 0,353002 | 0 | 74 | 50 | 3491 | under |
| [GO:0005509](../../../../gossipinfo/GO/0005509) | calcium ion binding | 0 | 0 | 0,353002 | 0 | 74 | 50 | 3491 | under |
| [GO:0005783](../../../../gossipinfo/GO/0005783) | endoplasmic reticulum | 0 | 0 | 0,358056 | 0 | 73 | 50 | 3492 | under |
| [GO:0034622](../../../../gossipinfo/GO/0034622) | cellular macromolecular complex assembly | 0 | 0 | 0,368379 | 0 | 71 | 50 | 3494 | under |
| [GO:0010604](../../../../gossipinfo/GO/0010604) | positive regulation of macromolecule metabolic process | 0 | 0 | 0,378993 | 0 | 69 | 50 | 3496 | under |
| [GO:0031325](../../../../gossipinfo/GO/0031325) | positive regulation of cellular metabolic process | 0 | 0 | 0,378993 | 0 | 69 | 50 | 3496 | under |
| [GO:0048598](../../../../gossipinfo/GO/0048598) | embryonic morphogenesis | 0 | 0 | 0,384412 | 0 | 68 | 50 | 3497 | under |
| [GO:0009055](../../../../gossipinfo/GO/0009055) | electron carrier activity | 0 | 0 | 0,395478 | 0 | 66 | 50 | 3499 | under |
| [GO:0030030](../../../../gossipinfo/GO/0030030) | cell projection organization | 0 | 0 | 0,395478 | 0 | 66 | 50 | 3499 | under |
| [GO:0016023](../../../../gossipinfo/GO/0016023) | cytoplasmic membrane-bounded vesicle | 0 | 0 | 0,395478 | 0 | 66 | 50 | 3499 | under |
| [GO:0008134](../../../../gossipinfo/GO/0008134) | transcription factor binding | 0 | 0 | 0,401128 | 0 | 65 | 50 | 3500 | under |
| [GO:0003700](../../../../gossipinfo/GO/0003700) | transcription factor activity | 0 | 0 | 0,401128 | 0 | 65 | 50 | 3500 | under |
| [GO:0016477](../../../../gossipinfo/GO/0016477) | cell migration | 0 | 0 | 0,406857 | 0 | 64 | 50 | 3501 | under |
| [GO:0010033](../../../../gossipinfo/GO/0010033) | response to organic substance | 0 | 0 | 0,406857 | 0 | 64 | 50 | 3501 | under |
| [GO:0005829](../../../../gossipinfo/GO/0005829) | cytosol | 0 | 0 | 0,408693 | 1 | 141 | 49 | 3424 | under |
| [GO:0050662](../../../../gossipinfo/GO/0050662) | coenzyme binding | 0 | 0 | 0,418556 | 0 | 62 | 50 | 3503 | under |
| [GO:0008135](../../../../gossipinfo/GO/0008135) | translation factor activity, nucleic acid binding | 0 | 0 | 0,424528 | 0 | 61 | 50 | 3504 | under |
| [GO:0070011](../../../../gossipinfo/GO/0070011) | peptidase activity, acting on L-amino acid peptides | 0 | 0 | 0,436671 | 1 | 134 | 49 | 3431 | under |
| [GO:0051172](../../../../gossipinfo/GO/0051172) | negative regulation of nitrogen compound metabolic process | 0 | 0 | 0,436725 | 0 | 59 | 50 | 3506 | under |
| [GO:0032787](../../../../gossipinfo/GO/0032787) | monocarboxylic acid metabolic process | 0 | 0 | 0,436725 | 0 | 59 | 50 | 3506 | under |
| [GO:0002009](../../../../gossipinfo/GO/0002009) | morphogenesis of an epithelium | 0 | 0 | 0,436725 | 0 | 59 | 50 | 3506 | under |
| [GO:0015672](../../../../gossipinfo/GO/0015672) | monovalent inorganic cation transport | 0 | 0 | 0,442951 | 0 | 58 | 50 | 3507 | under |
| [GO:0030054](../../../../gossipinfo/GO/0030054) | cell junction | 0 | 0 | 0,449265 | 0 | 57 | 50 | 3508 | under |
| [GO:0019318](../../../../gossipinfo/GO/0019318) | hexose metabolic process | 0 | 0 | 0,455667 | 0 | 56 | 50 | 3509 | under |
| [GO:0006091](../../../../gossipinfo/GO/0006091) | generation of precursor metabolites and energy | 0 | 0 | 0,462158 | 0 | 55 | 50 | 3510 | under |
| [GO:0006511](../../../../gossipinfo/GO/0006511) | ubiquitin-dependent protein catabolic process | 0 | 0 | 0,462158 | 0 | 55 | 50 | 3510 | under |
| [GO:0030001](../../../../gossipinfo/GO/0030001) | metal ion transport | 0 | 0 | 0,462158 | 0 | 55 | 50 | 3510 | under |
| [GO:0016481](../../../../gossipinfo/GO/0016481) | negative regulation of transcription | 0 | 0 | 0,462158 | 0 | 55 | 50 | 3510 | under |
| [GO:0006732](../../../../gossipinfo/GO/0006732) | coenzyme metabolic process | 0 | 0 | 0,468739 | 0 | 54 | 50 | 3511 | under |
| [GO:0007626](../../../../gossipinfo/GO/0007626) | locomotory behavior | 0 | 0 | 0,468739 | 0 | 54 | 50 | 3511 | under |
| [GO:0006164](../../../../gossipinfo/GO/0006164) | purine nucleotide biosynthetic process | 0 | 0 | 0,468739 | 0 | 54 | 50 | 3511 | under |
| [GO:0044430](../../../../gossipinfo/GO/0044430) | cytoskeletal part | 0 | 0 | 0,470225 | 1 | 126 | 49 | 3439 | under |
| [GO:0009260](../../../../gossipinfo/GO/0009260) | ribonucleotide biosynthetic process | 0 | 0 | 0,475412 | 0 | 53 | 50 | 3512 | under |
| [GO:0005216](../../../../gossipinfo/GO/0005216) | ion channel activity | 0 | 0 | 0,475412 | 0 | 53 | 50 | 3512 | under |
| [GO:0032268](../../../../gossipinfo/GO/0032268) | regulation of cellular protein metabolic process | 0 | 0 | 0,475412 | 0 | 53 | 50 | 3512 | under |
| [GO:0016874](../../../../gossipinfo/GO/0016874) | ligase activity | 0 | 0 | 0,478872 | 1 | 124 | 49 | 3441 | under |
| [GO:0008415](../../../../gossipinfo/GO/0008415) | acyltransferase activity | 0 | 0 | 0,482179 | 0 | 52 | 50 | 3513 | under |
| [GO:0042995](../../../../gossipinfo/GO/0042995) | cell projection | 0 | 0 | 0,482179 | 0 | 52 | 50 | 3513 | under |
| [GO:0022604](../../../../gossipinfo/GO/0022604) | regulation of cell morphogenesis | 0 | 0 | 0,482179 | 0 | 52 | 50 | 3513 | under |
| [GO:0007264](../../../../gossipinfo/GO/0007264) | small GTPase mediated signal transduction | 0 | 0 | 0,482179 | 0 | 52 | 50 | 3513 | under |
| [GO:0005626](../../../../gossipinfo/GO/0005626) | insoluble fraction | 0 | 0 | 0,482179 | 0 | 52 | 50 | 3513 | under |
| [GO:0042592](../../../../gossipinfo/GO/0042592) | homeostatic process | 0 | 0 | 0,48904 | 0 | 51 | 50 | 3514 | under |
| [GO:0003729](../../../../gossipinfo/GO/0003729) | mRNA binding | 0 | 0 | 0,48904 | 0 | 51 | 50 | 3514 | under |
| [GO:0003688](../../../../gossipinfo/GO/0003688) | DNA replication origin binding | 4,43E-008 | 1,11E-008 | 0 | 7 | 2 | 43 | 3563 | over |
| [GO:0006260](../../../../gossipinfo/GO/0006260) | DNA replication | 1,10E-007 | 8,23E-008 | 4,63E-010 | 8 | 16 | 42 | 3549 | over |
| [GO:0044419](../../../../gossipinfo/GO/0044419) | interspecies interaction between organisms | 1,56E-007 | 1,56E-007 | 1,10E-009 | 7 | 10 | 43 | 3555 | over |
| [GO:0031072](../../../../gossipinfo/GO/0031072) | heat shock protein binding | 1,62E-006 | 2,03E-006 | 1,82E-008 | 7 | 17 | 43 | 3548 | over |
| [GO:0019732](../../../../gossipinfo/GO/0019732) | antifungal humoral response | 2,04E-006 | 6,11E-006 | 3,24E-008 | 4 | 0 | 46 | 3565 | over |
| [GO:0019855](../../../../gossipinfo/GO/0019855) | calcium channel inhibitor activity | 2,04E-006 | 6,11E-006 | 3,24E-008 | 4 | 0 | 46 | 3565 | over |
| [GO:0006805](../../../../gossipinfo/GO/0006805) | xenobiotic metabolic process | 8,08E-006 | 2,83E-005 | 1,60E-007 | 4 | 1 | 46 | 3564 | over |
| [GO:0045087](../../../../gossipinfo/GO/0045087) | innate immune response | 2,98E-004 | 0,00119228 | 6,40E-006 | 4 | 6 | 46 | 3559 | over |
| [GO:0004386](../../../../gossipinfo/GO/0004386) | helicase activity | 5,96E-004 | 0,00267727 | 1,39E-005 | 7 | 53 | 43 | 3512 | over |
| [GO:0042742](../../../../gossipinfo/GO/0042742) | defense response to bacterium | 7,21E-004 | 0,003601 | 2,11E-005 | 4 | 9 | 46 | 3556 | over |
| [GO:0008270](../../../../gossipinfo/GO/0008270) | zinc ion binding | 0,0125836 | 0,0698014 | 2,25E-004 | 10 | 182 | 40 | 3383 | over |
| [GO:0016568](../../../../gossipinfo/GO/0016568) | chromatin modification | 0,0141713 | 0,484151 | 0,495996 | 0 | 50 | 50 | 3515 | under |
| [GO:0003779](../../../../gossipinfo/GO/0003779) | actin binding | 0,0141713 | 0,484151 | 0,495996 | 0 | 50 | 50 | 3515 | under |
| [GO:0032990](../../../../gossipinfo/GO/0032990) | cell part morphogenesis | 0,0141713 | 0,484151 | 0,495996 | 0 | 50 | 50 | 3515 | under |
| [GO:0006355](../../../../gossipinfo/GO/0006355) | regulation of transcription, DNA-dependent | 0,016978 | 0,100679 | 4,69E-004 | 9 | 163 | 41 | 3402 | over |
| [GO:0009150](../../../../gossipinfo/GO/0009150) | purine ribonucleotide metabolic process | 0,0222856 | 0,655324 | 0,50305 | 0 | 49 | 50 | 3516 | under |
| [GO:0016614](../../../../gossipinfo/GO/0016614) | oxidoreductase activity, acting on CH-OH group of donors | 0,0222856 | 0,655324 | 0,50305 | 0 | 49 | 50 | 3516 | under |
| [GO:0043085](../../../../gossipinfo/GO/0043085) | positive regulation of catalytic activity | 0,0222856 | 0,655324 | 0,50305 | 0 | 49 | 50 | 3516 | under |
| [GO:0044271](../../../../gossipinfo/GO/0044271) | cellular nitrogen compound biosynthetic process | 0,0222856 | 0,655324 | 0,505414 | 1 | 118 | 49 | 3447 | under |
| [GO:0007348](../../../../gossipinfo/GO/0007348) | regulation of syncytial blastoderm mitotic cell cycle | 0,0262679 | 0,167967 | 5,58E-004 | 2 | 1 | 48 | 3564 | over |
| [GO:0008630](../../../../gossipinfo/GO/0008630) | DNA damage response, signal transduction resulting in induction of apoptosis | 0,0262679 | 0,167967 | 5,58E-004 | 2 | 1 | 48 | 3564 | over |
| [GO:0016820](../../../../gossipinfo/GO/0016820) | hydrolase activity, acting on acid anhydrides, catalyzing transmembrane movement of substances | 0,0325942 | 0,800302 | 0,510201 | 0 | 48 | 50 | 3517 | under |
| [GO:0022890](../../../../gossipinfo/GO/0022890) | inorganic cation transmembrane transporter activity | 0,0325942 | 0,800302 | 0,510201 | 0 | 48 | 50 | 3517 | under |
| [GO:0007155](../../../../gossipinfo/GO/0007155) | cell adhesion | 0,0325942 | 0,800302 | 0,510201 | 0 | 48 | 50 | 3517 | under |
| [GO:0055114](../../../../gossipinfo/GO/0055114) | oxidation reduction | 0,0325942 | 0,800302 | 0,514458 | 1 | 116 | 49 | 3449 | under |
| [GO:0031224](../../../../gossipinfo/GO/0031224) | intrinsic to membrane | 0,0325942 | 0,800302 | 0,51668 | 3 | 255 | 47 | 3310 | under |
| [GO:0031572](../../../../gossipinfo/GO/0031572) | G2/M transition DNA damage checkpoint | 0,039053 | 0,282497 | 0,00110534 | 2 | 2 | 48 | 3563 | over |
