# Supplementary material for: An Expressed Sequence Tag collection from the male antennae of the Noctuid moth Spodoptera littoralis: a resource for olfactory and pheromone detection research
Source: BMC Genomics. 2011 Jan 29;12:86. doi: 10.1186/1471-2164-12-86 (PMC3045336; doi:10.1186/1471-2164-12-86)
Supplement: Additional file 4 — Table of the Interproscan results for the 678 S. littoralis unigene sequences having no similarity with any known proteins. [file 1471-2164-12-86-S4.DOC]

| id | Analysis | Domain Entry | Domain description | start | end | evalue | Interpro Entry | Interpro description |
| --- | --- | --- | --- | --- | --- | --- | --- | --- |
| FQ029394 | HMMPfam | PF00098 | zf-CCHC | 122 | 139 | 0.00041 | IPR001878 | Zinc finger, CCHC-type |
| FQ029394 | ProfileScan | PS50158 | ZF_CCHC | 123 | 138 | 9.455 | IPR001878 | Zinc finger, CCHC-type |
| FQ022373 | BlastProDom | PD968187 | Q30KS2_CANFA_Q30KS2; | 31 | 115 | 0.009 | NULL | NULL |
| FQ028309 | HMMPanther | PTHR23181 | RHO GTPASE ACTIVATING PROTEIN | 61 | 191 | 2.8e-09 | IPR015767 | Rho GTPase activating |
| FQ028309 | superfamily | SSF50729 | PH domain-like | 127 | 177 | 5.5e-06 | NULL | NULL |
| HO118406 | HMMPfam | PF00144 | Beta-lactamase | 31 | 118 | 6.4e-11 | IPR001466 | Beta-lactamase-related |
| HO118406 | superfamily | SSF56601 | beta-lactamase/transpeptidase-like | 24 | 122 | 4.3e-22 | IPR012338 | Beta-lactamase-type transpeptidase fold |
| HO118406 | PatternScan | PS00146 | BETA_LACTAMASE_A | 64 | 79 | NA | IPR000871 | Beta-lactamase, class A/D |
| FQ027164 | ProfileScan | PS50287 | SRCR_2 | 142 | 164 | 8.369 | IPR001190 | Speract/scavenger receptor |
| FQ027164 | ProfileScan | PS50835 | IG_LIKE | 1 | 86 | 7.286 | IPR007110 | Immunoglobulin-like |
| FQ021442 | HMMPfam | PF04500 | FLYWCH | 32 | 94 | 1.1e-09 | IPR007588 | Zinc finger, FLYWCH-type |
| FQ017015 | ProfileScan | PS50097 | BTB | 101 | 160 | 9.103 | IPR000210 | BTB/POZ-like |
| FQ017015 | HMMSmart | SM00225 | no description | 101 | 196 | 0.00031 | IPR000210 | BTB/POZ-like |
| FQ017015 | HMMPfam | PF00651 | BTB | 91 | 196 | 1.7e-09 | IPR013069 | BTB/POZ |
| FQ017015 | superfamily | SSF54695 | POZ domain | 76 | 195 | 1.2e-14 | IPR011333 | BTB/POZ fold |
| FQ017015 | HMMPanther | PTHR23230 | KELCH-RELATED PROTEIN | 111 | 239 | 1.7e-13 | IPR013089 | Kelch related |
| FQ017015 | Gene3D | G3DSA:3.30.710.10 | no description | 88 | 192 | 5.8e-10 | IPR011333 | BTB/POZ fold |
| FQ020630 | HMMPanther | PTHR20975 | FAMILY NOT NAMED | 62 | 154 | 9.1e-05 | NULL | NULL |
| FQ020630 | superfamily | SSF47565 | Insect pheromone/odorant-binding proteins | 79 | 151 | 5.6e-06 | IPR006170 | Pheromone/general odorant binding protein, PBP/GOBP |
| FQ016130 | PatternScan | PS00430 | TONB_DEPENDENT_REC_1 | 1 | 104 | NA | IPR010916 | TonB box, conserved site |
| FQ017728 | ProfileScan | PS51257 | PROKAR_LIPOPROTEIN | 1 | 20 | 5.000 | NULL | NULL |
| CL987Contig1 | BlastProDom | PD353114 | Q66I79_BRARE_Q66I79; | 1 | 46 | 0.0009 | NULL | NULL |
| FQ021057 | HMMPanther | PTHR21545:SF10 | TRANSCRIPTION FACTOR MLR1 | 5 | 44 | 9.2e-06 | NULL | NULL |
| FQ021057 | HMMPanther | PTHR21545 | TRANSCRIPTION FACTOR MLR1/2 | 5 | 44 | 9.2e-06 | NULL | NULL |
| HO118308 | HMMPfam | PF02380 | Papo_T_antigen | 137 | 160 | 1.1e-08 | IPR003354 | Small/middle T-antigen |
| HO118350 | superfamily | SSF56601 | beta-lactamase/transpeptidase-like | 1 | 67 | 5.9e-06 | IPR012338 | Beta-lactamase-type transpeptidase fold |
| CL1Contig693 | HMMPanther | PTHR23080 | THAP DOMAIN PROTEIN | 33 | 93 | 1.7e-05 | NULL | NULL |
| CL1Contig693 | ProfileScan | PS50950 | THAP | 11 | 94 | 13.107 | IPR006612 | Zinc finger, C2CH-type |
| CL1Contig693 | HMMPfam | PF05485 | THAP | 27 | 100 | 2.7e-07 | IPR006612 | Zinc finger, C2CH-type |
| FQ032656 | PatternScan | PS00584 | PFKB_KINASES_2 | 80 | 93 | NA | IPR002173 | Carbohydrate/puine kinase, PfkB, conserved site |
| HO118312 | superfamily | SSF56601 | beta-lactamase/transpeptidase-like | 24 | 129 | 2.8e-27 | IPR012338 | Beta-lactamase-type transpeptidase fold |
| HO118312 | HMMPfam | PF00144 | Beta-lactamase | 31 | 133 | 6.4e-10 | IPR001466 | Beta-lactamase-related |
| HO118312 | Gene3D | G3DSA:3.40.710.10 | no description | 44 | 129 | 0.001 | NULL | NULL |
| HO118312 | PatternScan | PS00146 | BETA_LACTAMASE_A | 64 | 79 | NA | IPR000871 | Beta-lactamase, class A/D |
| FQ020289 | PatternScan | PS00284 | SERPIN | 34 | 44 | NA | IPR000215 | Protease inhibitor I4, serpin |
| FQ031073 | HMMPfam | PF08725 | Integrin_b_cyt | 37 | 56 | 3.1e-08 | IPR014836 | Integrin beta subunit, cytoplasmic |
| FQ031073 | HMMPanther | PTHR10082 | INTEGRIN BETA SUBUNIT | 37 | 55 | 0.00075 | IPR001169 | Integrin beta subunit, C-terminal |
| FQ018236 | HMMPanther | PTHR19959:SF20 | KINESIN LIGHT CHAIN 1, 2, 8 | 24 | 49 | 1.9e-07 | NULL | NULL |
| FQ018236 | HMMPanther | PTHR19959 | KINESIN LIGHT CHAIN | 24 | 49 | 1.9e-07 | NULL | NULL |
| FQ031135 | ProfileScan | PS51257 | PROKAR_LIPOPROTEIN | 1 | 26 | 5.000 | NULL | NULL |
| FQ020683 | HMMPanther | PTHR10489:SF39 | IMMUNOGLOBULIN DOMAIN SUPERFAMILY (SENSORY GUIDANCE PROTEIN) | 28 | 53 | 4.3e-06 | NULL | NULL |
| FQ020683 | HMMPanther | PTHR10489 | CELL ADHESION MOLECULE | 28 | 53 | 4.3e-06 | NULL | NULL |
| FQ025255 | HMMPanther | PTHR11227 | WD-REPEAT PROTEIN INTERACTING WITH PHOSPHOINOSIDES (WIPI)-RELATED | 1 | 37 | 0.00079 | NULL | NULL |
| HO118375 | HMMPanther | PTHR10066:SF5 | BETA-GALACTOSIDASE | 16 | 34 | 2.5e-09 | NULL | NULL |
| HO118375 | HMMPanther | PTHR10066 | GLYCOSIDE HYDROLASE | 16 | 34 | 2.5e-09 | NULL | NULL |
| FQ021723 | HMMPanther | PTHR11685:SF5 | RING FINGER PROTEIN 19 (DORFIN) (DOUBLE RING-FINGER PROTEIN) | 24 | 66 | 1.5e-13 | NULL | NULL |
| FQ021723 | HMMPanther | PTHR11685 | ARIADNE RING ZINC FINGER | 24 | 66 | 1.5e-13 | NULL | NULL |
