# Supplementary material for: An Expressed Sequence Tag collection from the male antennae of the Noctuid moth Spodoptera littoralis: a resource for olfactory and pheromone detection research
Source: BMC Genomics. 2011 Jan 29;12:86. doi: 10.1186/1471-2164-12-86 (PMC3045336; doi:10.1186/1471-2164-12-86)
Supplement: Additional file 5 — Table of the GO terms over or under represented in S. littoralis unigenes having no lepidopteran EST match. Test group: S. littoralis ESTs with no match. Reference group: S. littoralis ESTs with at least one match. Fisher's exact test with multiple testing correction. [file 1471-2164-12-86-S5.DOC]

| **GO Term** | **Name** | **FDR** | **FWER** | **single test p-Value** | **# in test group** | **# in reference group** | **# non annot test** | **# non annot reference group** | **Over/Under** |
| --- | --- | --- | --- | --- | --- | --- | --- | --- | --- |
| [GO:0005811](../../../../gossipinfo/GO/0005811) | lipid particle | 2,48E-007 | 1,67E-006 | 4,14E-008 | 13 | 155 | 836 | 2611 | under |
| [GO:0005525](../../../../gossipinfo/GO/0005525) | GTP binding | 3,50E-007 | 2,62E-006 | 7,18E-008 | 5 | 103 | 844 | 2663 | under |
| [GO:0003924](../../../../gossipinfo/GO/0003924) | GTPase activity | 9,02E-007 | 7,44E-006 | 2,14E-007 | 2 | 76 | 847 | 2690 | under |
| [GO:0005549](../../../../gossipinfo/GO/0005549) | odorant binding | 5,13E-006 | 2,57E-006 | 2,51E-008 | 22 | 9 | 827 | 2757 | over |
| [GO:0006457](../../../../gossipinfo/GO/0006457) | protein folding | 1,69E-005 | 1,69E-004 | 4,11E-006 | 0 | 46 | 849 | 2720 | under |
| [GO:0005524](../../../../gossipinfo/GO/0005524) | ATP binding | 2,51E-005 | 2,64E-004 | 6,12E-006 | 43 | 269 | 806 | 2497 | under |
| [GO:0001883](../../../../gossipinfo/GO/0001883) | purine nucleoside binding | 7,49E-005 | 9,73E-004 | 1,89E-005 | 50 | 290 | 799 | 2476 | under |
| [GO:0004984](../../../../gossipinfo/GO/0004984) | olfactory receptor activity | 7,99E-005 | 7,99E-005 | 4,90E-007 | 10 | 0 | 839 | 2766 | over |
| [GO:0000381](../../../../gossipinfo/GO/0000381) | regulation of alternative nuclear mRNA splicing, via spliceosome | 9,88E-005 | 0,00138173 | 2,74E-005 | 0 | 39 | 849 | 2727 | under |
| [GO:0051082](../../../../gossipinfo/GO/0051082) | unfolded protein binding | 1,23E-004 | 0,00186666 | 3,60E-005 | 0 | 38 | 849 | 2728 | under |
| [GO:0003735](../../../../gossipinfo/GO/0003735) | structural constituent of ribosome | 1,23E-004 | 0,00186666 | 3,60E-005 | 0 | 38 | 849 | 2728 | under |
| [GO:0003743](../../../../gossipinfo/GO/0003743) | translation initiation factor activity | 3,71E-004 | 0,00637598 | 1,06E-004 | 0 | 34 | 849 | 2732 | under |
| [GO:0003729](../../../../gossipinfo/GO/0003729) | mRNA binding | 5,05E-004 | 0,00938134 | 1,49E-004 | 2 | 49 | 847 | 2717 | under |
| [GO:0055114](../../../../gossipinfo/GO/0055114) | oxidation reduction | 5,52E-004 | 0,010434 | 1,70E-004 | 12 | 105 | 837 | 2661 | under |
| [GO:0016616](../../../../gossipinfo/GO/0016616) | oxidoreductase activity, acting on the CH-OH group of donors, NAD or NADP as acceptor | 8,46E-004 | 0,0178233 | 2,82E-004 | 1 | 39 | 848 | 2727 | under |
| [GO:0042325](../../../../gossipinfo/GO/0042325) | regulation of phosphorylation | 8,46E-004 | 0,0178233 | 2,82E-004 | 1 | 39 | 848 | 2727 | under |
| [GO:0005759](../../../../gossipinfo/GO/0005759) | mitochondrial matrix | 0,00172458 | 0,0416134 | 5,90E-004 | 1 | 36 | 848 | 2730 | under |
| [GO:0007264](../../../../gossipinfo/GO/0007264) | small GTPase mediated signal transduction | 0,00189966 | 0,0463839 | 6,59E-004 | 3 | 49 | 846 | 2717 | under |
| [GO:0043436](../../../../gossipinfo/GO/0043436) | oxoacid metabolic process | 0,00230449 | 0,0603288 | 7,56E-004 | 18 | 124 | 831 | 2642 | under |
| [GO:0044271](../../../../gossipinfo/GO/0044271) | cellular nitrogen compound biosynthetic process | 0,00243972 | 0,0648956 | 8,23E-004 | 14 | 105 | 835 | 2661 | under |
| [GO:0034404](../../../../gossipinfo/GO/0034404) | nucleobase, nucleoside and nucleotide biosynthetic process | 0,00282953 | 0,0820393 | 0,00101896 | 6 | 64 | 843 | 2702 | under |
| [GO:0044455](../../../../gossipinfo/GO/0044455) | mitochondrial membrane part | 0,00318066 | 0,0967821 | 0,00120914 | 0 | 25 | 849 | 2741 | under |
| [GO:0007417](../../../../gossipinfo/GO/0007417) | central nervous system development | 0,00318066 | 0,0967821 | 0,00120914 | 0 | 25 | 849 | 2741 | under |
| [GO:0005509](../../../../gossipinfo/GO/0005509) | calcium ion binding | 0,00367245 | 0,118206 | 0,0015412 | 7 | 67 | 842 | 2699 | under |
| [GO:0006259](../../../../gossipinfo/GO/0006259) | DNA metabolic process | 0,00401326 | 0,00500403 | 3,06E-005 | 31 | 36 | 818 | 2730 | over |
| [GO:0008026](../../../../gossipinfo/GO/0008026) | ATP-dependent helicase activity | 0,00437542 | 0,148556 | 0,00186142 | 2 | 38 | 847 | 2728 | under |
| [GO:0006468](../../../../gossipinfo/GO/0006468) | protein amino acid phosphorylation | 0,00448838 | 0,153036 | 0,00195932 | 12 | 90 | 837 | 2676 | under |
| [GO:0006099](../../../../gossipinfo/GO/0006099) | tricarboxylic acid cycle | 0,00477436 | 0,169919 | 0,00207365 | 0 | 23 | 849 | 2743 | under |
| [GO:0033279](../../../../gossipinfo/GO/0033279) | ribosomal subunit | 0,00611108 | 0,222864 | 0,00271524 | 0 | 22 | 849 | 2744 | under |
| [GO:0007391](../../../../gossipinfo/GO/0007391) | dorsal closure | 0,00611108 | 0,222864 | 0,00271524 | 0 | 22 | 849 | 2744 | under |
| [GO:0008553](../../../../gossipinfo/GO/0008553) | hydrogen-exporting ATPase activity, phosphorylative mechanism | 0,00762187 | 0,289069 | 0,00355503 | 0 | 21 | 849 | 2745 | under |
| [GO:0046483](../../../../gossipinfo/GO/0046483) | heterocycle metabolic process | 0,00829266 | 0,315806 | 0,00390737 | 13 | 90 | 836 | 2676 | under |
| [GO:0015986](../../../../gossipinfo/GO/0015986) | ATP synthesis coupled proton transport | 0,00868869 | 0,332466 | 0,00410371 | 1 | 28 | 848 | 2738 | under |
| [GO:0016853](../../../../gossipinfo/GO/0016853) | isomerase activity | 0,00955157 | 0,369376 | 0,00451432 | 2 | 34 | 847 | 2732 | under |
| [GO:0045860](../../../../gossipinfo/GO/0045860) | positive regulation of protein kinase activity | 0,0100469 | 0,391968 | 0,00465417 | 0 | 20 | 849 | 2746 | under |
| [GO:0015031](../../../../gossipinfo/GO/0015031) | protein transport | 0,0101692 | 0,400235 | 0,00494257 | 17 | 106 | 832 | 2660 | under |
| [GO:0035091](../../../../gossipinfo/GO/0035091) | phosphoinositide binding | 0,0125088 | 0,481277 | 0,00609261 | 0 | 19 | 849 | 2747 | under |
| [GO:0033178](../../../../gossipinfo/GO/0033178) | proton-transporting two-sector ATPase complex, catalytic domain | 0,0125088 | 0,481277 | 0,00609261 | 0 | 19 | 849 | 2747 | under |
| [GO:0006096](../../../../gossipinfo/GO/0006096) | glycolysis | 0,0125088 | 0,481277 | 0,00609261 | 0 | 19 | 849 | 2747 | under |
| [GO:0006414](../../../../gossipinfo/GO/0006414) | translational elongation | 0,0125088 | 0,481277 | 0,00609261 | 0 | 19 | 849 | 2747 | under |
| [GO:0051240](../../../../gossipinfo/GO/0051240) | positive regulation of multicellular organismal process | 0,0125088 | 0,481277 | 0,00609261 | 0 | 19 | 849 | 2747 | under |
| [GO:0000022](../../../../gossipinfo/GO/0000022) | mitotic spindle elongation | 0,0125088 | 0,481277 | 0,00609261 | 0 | 19 | 849 | 2747 | under |
| [GO:0008360](../../../../gossipinfo/GO/0008360) | regulation of cell shape | 0,0137915 | 0,531584 | 0,0066026 | 3 | 38 | 846 | 2728 | under |
| [GO:0043623](../../../../gossipinfo/GO/0043623) | cellular protein complex assembly | 0,0137915 | 0,531584 | 0,0066026 | 3 | 38 | 846 | 2728 | under |
| [GO:0060341](../../../../gossipinfo/GO/0060341) | regulation of cellular localization | 0,0141391 | 0,545581 | 0,00697375 | 2 | 32 | 847 | 2734 | under |
| [GO:0016251](../../../../gossipinfo/GO/0016251) | general RNA polymerase II transcription factor activity | 0,0141391 | 0,545581 | 0,00697375 | 2 | 32 | 847 | 2734 | under |
| [GO:0008134](../../../../gossipinfo/GO/0008134) | transcription factor binding | 0,0143156 | 0,554847 | 0,00729696 | 7 | 58 | 842 | 2708 | under |
| [GO:0030036](../../../../gossipinfo/GO/0030036) | actin cytoskeleton organization | 0,0148453 | 0,570722 | 0,00786431 | 9 | 67 | 840 | 2699 | under |
| [GO:0030532](../../../../gossipinfo/GO/0030532) | small nuclear ribonucleoprotein complex | 0,0203237 | 0,703415 | 0,0104379 | 0 | 17 | 849 | 2749 | under |
| [GO:0007298](../../../../gossipinfo/GO/0007298) | border follicle cell migration | 0,0210259 | 0,725092 | 0,0105687 | 1 | 24 | 848 | 2742 | under |
| [GO:0003746](../../../../gossipinfo/GO/0003746) | translation elongation factor activity | 0,0210259 | 0,725092 | 0,0105687 | 1 | 24 | 848 | 2742 | under |
| [GO:0005730](../../../../gossipinfo/GO/0005730) | nucleolus | 0,0210259 | 0,728294 | 0,010598 | 5 | 46 | 844 | 2720 | under |
| [GO:0007422](../../../../gossipinfo/GO/0007422) | peripheral nervous system development | 0,0253011 | 0,802484 | 0,0133465 | 1 | 23 | 848 | 2743 | under |
| [GO:0007608](../../../../gossipinfo/GO/0007608) | sensory perception of smell | 0,0253194 | 0,0553775 | 2,49E-004 | 12 | 7 | 837 | 2759 | over |
| [GO:0004767](../../../../gossipinfo/GO/0004767) | sphingomyelin phosphodiesterase activity | 0,0265232 | 0,822019 | 0,0136604 | 0 | 16 | 849 | 2750 | under |
| [GO:0046961](../../../../gossipinfo/GO/0046961) | proton-transporting ATPase activity, rotational mechanism | 0,0265232 | 0,822019 | 0,0136604 | 0 | 16 | 849 | 2750 | under |
| [GO:0051173](../../../../gossipinfo/GO/0051173) | positive regulation of nitrogen compound metabolic process | 0,0278737 | 0,848011 | 0,0150991 | 4 | 39 | 845 | 2727 | under |
| [GO:0040007](../../../../gossipinfo/GO/0040007) | growth | 0,0278737 | 0,848011 | 0,0150991 | 4 | 39 | 845 | 2727 | under |
| [GO:0006520](../../../../gossipinfo/GO/0006520) | cellular amino acid metabolic process | 0,0282044 | 0,852664 | 0,0154426 | 10 | 67 | 839 | 2699 | under |
| [GO:0048813](../../../../gossipinfo/GO/0048813) | dendrite morphogenesis | 0,0286977 | 0,865037 | 0,01633 | 2 | 28 | 847 | 2738 | under |
| [GO:0006901](../../../../gossipinfo/GO/0006901) | vesicle coating | 0,0300369 | 0,878233 | 0,0168302 | 1 | 22 | 848 | 2744 | under |
| [GO:0045944](../../../../gossipinfo/GO/0045944) | positive regulation of transcription from RNA polymerase II promoter | 0,0327886 | 0,912705 | 0,0178762 | 0 | 15 | 849 | 2751 | under |
| [GO:0045475](../../../../gossipinfo/GO/0045475) | locomotor rhythm | 0,0327886 | 0,912705 | 0,0178762 | 0 | 15 | 849 | 2751 | under |
| [GO:0006413](../../../../gossipinfo/GO/0006413) | translational initiation | 0,0327886 | 0,912705 | 0,0178762 | 0 | 15 | 849 | 2751 | under |
| [GO:0005200](../../../../gossipinfo/GO/0005200) | structural constituent of cytoskeleton | 0,0327886 | 0,912705 | 0,0178762 | 0 | 15 | 849 | 2751 | under |
| [GO:0006090](../../../../gossipinfo/GO/0006090) | pyruvate metabolic process | 0,0327886 | 0,912705 | 0,0178762 | 0 | 15 | 849 | 2751 | under |
| [GO:0051437](../../../../gossipinfo/GO/0051437) | positive regulation of ubiquitin-protein ligase activity during mitotic cell cycle | 0,0327886 | 0,912705 | 0,0178762 | 0 | 15 | 849 | 2751 | under |
| [GO:0005832](../../../../gossipinfo/GO/0005832) | chaperonin-containing T-complex | 0,0327886 | 0,912705 | 0,0178762 | 0 | 15 | 849 | 2751 | under |
| [GO:0003700](../../../../gossipinfo/GO/0003700) | transcription factor activity | 0,0329175 | 0,91425 | 0,0179523 | 8 | 57 | 841 | 2709 | under |
| [GO:0051049](../../../../gossipinfo/GO/0051049) | regulation of transport | 0,0331143 | 0,9162 | 0,018106 | 4 | 38 | 845 | 2728 | under |
| [GO:0048037](../../../../gossipinfo/GO/0048037) | cofactor binding | 0,0339372 | 0,922548 | 0,0195719 | 12 | 74 | 837 | 2692 | under |
| [GO:0007269](../../../../gossipinfo/GO/0007269) | neurotransmitter secretion | 0,034726 | 0,930136 | 0,0201109 | 2 | 27 | 847 | 2739 | under |
| [GO:0007186](../../../../gossipinfo/GO/0007186) | G-protein coupled receptor protein signaling pathway | 0,0356465 | 0,0852635 | 4,50E-004 | 18 | 18 | 831 | 2748 | over |
| [GO:0070201](../../../../gossipinfo/GO/0070201) | regulation of establishment of protein localization | 0,037049 | 0,944199 | 0,0211907 | 1 | 21 | 848 | 2745 | under |
| [GO:0031344](../../../../gossipinfo/GO/0031344) | regulation of cell projection organization | 0,037049 | 0,944199 | 0,0211907 | 1 | 21 | 848 | 2745 | under |
| [GO:0008340](../../../../gossipinfo/GO/0008340) | determination of adult lifespan | 0,037049 | 0,944199 | 0,0211907 | 1 | 21 | 848 | 2745 | under |
| [GO:0019842](../../../../gossipinfo/GO/0019842) | vitamin binding | 0,0372206 | 0,946462 | 0,0214679 | 3 | 32 | 846 | 2734 | under |
| [GO:0019866](../../../../gossipinfo/GO/0019866) | organelle inner membrane | 0,0372206 | 0,946462 | 0,0214679 | 3 | 32 | 846 | 2734 | under |
| [GO:0046907](../../../../gossipinfo/GO/0046907) | intracellular transport | 0,0376999 | 0,950361 | 0,022066 | 27 | 134 | 822 | 2632 | under |
| [GO:0016831](../../../../gossipinfo/GO/0016831) | carboxy-lyase activity | 0,0398708 | 0,962973 | 0,023391 | 0 | 14 | 849 | 2752 | under |
| [GO:0051436](../../../../gossipinfo/GO/0051436) | negative regulation of ubiquitin-protein ligase activity during mitotic cell cycle | 0,0398708 | 0,962973 | 0,023391 | 0 | 14 | 849 | 2752 | under |
| [GO:0030120](../../../../gossipinfo/GO/0030120) | vesicle coat | 0,0398708 | 0,962973 | 0,023391 | 0 | 14 | 849 | 2752 | under |
| [GO:0043254](../../../../gossipinfo/GO/0043254) | regulation of protein complex assembly | 0,0398708 | 0,962973 | 0,023391 | 0 | 14 | 849 | 2752 | under |
| [GO:0042440](../../../../gossipinfo/GO/0042440) | pigment metabolic process | 0,0398708 | 0,962973 | 0,023391 | 0 | 14 | 849 | 2752 | under |
| [GO:0046328](../../../../gossipinfo/GO/0046328) | regulation of JNK cascade | 0,0398708 | 0,962973 | 0,023391 | 0 | 14 | 849 | 2752 | under |
| [GO:0030005](../../../../gossipinfo/GO/0030005) | cellular di-, tri-valent inorganic cation homeostasis | 0,0398708 | 0,962973 | 0,023391 | 0 | 14 | 849 | 2752 | under |
| [GO:0031145](../../../../gossipinfo/GO/0031145) | anaphase-promoting complex-dependent proteasomal ubiquitin-dependent protein catabolic process | 0,0398708 | 0,962973 | 0,023391 | 0 | 14 | 849 | 2752 | under |
| [GO:0070302](../../../../gossipinfo/GO/0070302) | regulation of stress-activated protein kinase signaling pathway | 0,0398708 | 0,962973 | 0,023391 | 0 | 14 | 849 | 2752 | under |
| [GO:0045664](../../../../gossipinfo/GO/0045664) | regulation of neuron differentiation | 0,0416328 | 0,968664 | 0,0247178 | 2 | 26 | 847 | 2740 | under |
| [GO:0004672](../../../../gossipinfo/GO/0004672) | protein kinase activity | 0,0419966 | 0,970547 | 0,0251274 | 20 | 105 | 829 | 2661 | under |
| [GO:0016874](../../../../gossipinfo/GO/0016874) | ligase activity | 0,0419966 | 0,970547 | 0,0251274 | 20 | 105 | 829 | 2661 | under |
| [GO:0009607](../../../../gossipinfo/GO/0009607) | response to biotic stimulus | 0,0427142 | 0,972569 | 0,025876 | 4 | 36 | 845 | 2730 | under |
| [GO:0016585](../../../../gossipinfo/GO/0016585) | chromatin remodeling complex | 0,0447617 | 0,978456 | 0,0266369 | 1 | 20 | 848 | 2746 | under |
| [GO:0031400](../../../../gossipinfo/GO/0031400) | negative regulation of protein modification process | 0,0447617 | 0,978456 | 0,0266369 | 1 | 20 | 848 | 2746 | under |
| [GO:0004252](../../../../gossipinfo/GO/0004252) | serine-type endopeptidase activity | 0,0447617 | 0,978456 | 0,0266369 | 1 | 20 | 848 | 2746 | under |
| [GO:0005789](../../../../gossipinfo/GO/0005789) | endoplasmic reticulum membrane | 0,0447617 | 0,978456 | 0,0266369 | 1 | 20 | 848 | 2746 | under |
| [GO:0031401](../../../../gossipinfo/GO/0031401) | positive regulation of protein modification process | 0,0447617 | 0,978456 | 0,0266369 | 1 | 20 | 848 | 2746 | under |
| [GO:0006911](../../../../gossipinfo/GO/0006911) | phagocytosis, engulfment | 0,0447617 | 0,978666 | 0,026689 | 14 | 80 | 835 | 2686 | under |
| [GO:0005576](../../../../gossipinfo/GO/0005576) | extracellular region | 0,0454192 | 0,980568 | 0,0279238 | 6 | 45 | 843 | 2721 | under |
| [GO:0008270](../../../../gossipinfo/GO/0008270) | zinc ion binding | 0,0458914 | 0,98194 | 0,0292415 | 34 | 158 | 815 | 2608 | under |
| [GO:0010608](../../../../gossipinfo/GO/0010608) | posttranscriptional regulation of gene expression | 0,0480475 | 0,986095 | 0,030316 | 2 | 25 | 847 | 2741 | under |
