# Supplementary material for: An Expressed Sequence Tag collection from the male antennae of the Noctuid moth Spodoptera littoralis: a resource for olfactory and pheromone detection research
Source: BMC Genomics. 2011 Jan 29;12:86. doi: 10.1186/1471-2164-12-86 (PMC3045336; doi:10.1186/1471-2164-12-86)
Supplement: Additional file 6 — Accession numbers for amino acid sequences of the lepidopteran odorant-binding proteins, antennal binding proteins, chemosensory proteins and olfactory receptors used in the phylogenetic analyses. [file 1471-2164-12-86-S6.DOC]

| **Protein name** | **Accession number** |
| --- | --- |
| BmorOR1 | BAD69584 |
| BmorOR2 | BAD69585 |
| BmorOR3 | BAD89567 |
| BmorOR4 | BAD89568 |
| BmorOR5 | BAD89569 |
| BmorOR6 | BAD89570 |
| BmorOR7 | CAI43913 |
| BmorOR8 | BAH66308 |
| BmorOR9 | BAH66309 |
| BmorOR10 | DAA05970 |
| BmorOR11 | BAH66310 |
| BmorOR12 | BAH66311 |
| BmorOR13 | BAH66312 |
| BmorOR14 | BAH66313 |
| BmorOR15 | DAA05974 |
| BmorOR16 | BAH66314 |
| BmorOR17 | BAH66315 |
| BmorOR18 | BAH66316 |
| BmorOR19 | DAA05977 |
| BmorOR20 | BAH66317 |
| BmorOR21 | BAH66318 |
| BmorOR22 | BAH66319 |
| BmorOR23 | BAH66320 |
| BmorOR24 | BAH66321 |
| BmorOR25 | BAH66322 |
| BmorOR26 | BAH66323 |
| BmorOR27 | BAH66324 |
| BmorOR28 | BAH66325 |
| BmorOR29 | BAH66326 |
| BmorOR30 | BAH66327 |
| BmorOR32 | BAH66328 |
| BmorOR33 | DAA05989 |
| BmorOR34 | DAA05990 |
| BmorOR35 | DAA05991 |
| BmorOR36 | BAH66333 |
| BmorOR37 | DAA05993 |
| BmorOR38 | DAA05994 |
| BmorOR39 | BAH66336 |
| BmorOR40 | BAH66337 |
| BmorOR41 | DAA05997 |
| BmorOR42 | BAH66338 |
| BmorOR44 | BAH66339 |
| BmorOR45 | DAA06001 |
| BmorOR46 | BAH66341 |
| BmorOR47 | DAA06003 |
| BmorOR50 | BAH66344+BAH66345 |
| BmorOR53 | BAH66347 |
|  |  |
|  |  |
| BmorOR54 | BAH66348 |
| BmorOR55 | BAH66349 |
| BmorOR56 | BAH66350 |
| BmorOR57 | BAH66351 |
| BmorOR58 | BAH66352 |
| BmorOR59 | BAH66353 |
| BmorOR60 | BAH66354 |
| BmorOR61 | BAH66355 |
| BmorOR62 | BAH66356+BAH66357 |
| BmorOR63 | BAH66358 |
| BmorOR64 | BAH66359 |
| DindOR1 | BAG71417 |
| DindOR2 | BAG71418 |
| DindOR3 | BAG71424 |
| EposOR1 | ACJ12927 |
| EposOR2 | ACJ12928 |
| EposOR3 | ACJ12929 |
| HvirOR1 | CAD31850 |
| HvirOR2 | CAD31851 |
| HvirOR3 | CAD31852 |
| HvirOR4 | CAD31946 |
| HvirOR5 | CAD31947 |
| HvirOR6 | CAD31948 |
| HvirOR7 | CAD31853 |
| HvirOR8 | CAD31949 |
| HvirOR9 | CAD31950 |
| HvirOR10 | CAG38111 |
| HvirOR11 | CAG38112 |
| HvirOR12 | CAG38113 |
| HvirOR13 | CAG38114 |
| HvirOR14 | CAG38115 |
| HvirOR15 | CAG38116 |
| HvirOR16 | CAG38117 |
| HvirOR17 | CAG38118 |
| HvirOR18 | CAG38119 |
| HvirOR19 | CAG38120 |
| HvirOR20 | CAG38121 |
| HvirOR21 | CAG38122 |
| MsepOR1 | BAG71714 |
| MsepOR2 | BAG71415 |
| MsepOR3 | BAG71423 |
| PxylOR1 | BAG71420 |
| PxylOR2 | BAG71421 |
| PxylOR3 | BAG71425 |
| PxylOR4 | BAG71426 |
|  |  |
|  |  |
|  |  |
|  |  |

| **Protein name** | **Accession number** |
| --- | --- |
| BmorOBP1 | Gong et al. 2009 |
| BmorOBP2 | Gong et al. 2009 |
| BmorOBP3 | Gong et al. 2009 |
| BmorOBP4 | Gong et al. 2009 |
| BmorOBP5 | Gong et al. 2009 |
| BmorOBP6 | Gong et al. 2009 |
| BmorOBP7 | Gong et al. 2009 |
| BmorOBP8 | Gong et al. 2009 |
| BmorOBP9 | Gong et al. 2009 |
| BmorOBP10 | Gong et al. 2009 |
| BmorOBP11 | Gong et al. 2009 |
| BmorOBP12 | Gong et al. 2009 |
| BmorOBP13 | Gong et al. 2009 |
| BmorOBP14 | Gong et al. 2009 |
| BmorOBP15 | Gong et al. 2009 |
| BmorOBP16 | Gong et al. 2009 |
| BmorOBP17 | Gong et al. 2009 |
| BmorOBP18 | Gong et al. 2009 |
| BmorOBP19 | Gong et al. 2009 |
| BmorOBP20 | Gong et al. 2009 |
| BmorOBP21 | Gong et al. 2009 |
| BmorOBP22 | Gong et al. 2009 |
| BmorOBP23 | Gong et al. 2009 |
| BmorOBP25 | Gong et al. 2009 |
| BmorOBP26 | Gong et al. 2009 |
| BmorOBP27 | Gong et al. 2009 |
| BmorOBP28 | Gong et al. 2009 |
| BmorOBP29 | Gong et al. 2009 |
| BmorOBP30 | Gong et al. 2009 |
| BmorOBP31 | Gong et al. 2009 |
| BmorOBP32 | Gong et al. 2009 |
| BmorOBP33 | Gong et al. 2009 |
| BmorOBP34 | Gong et al. 2009 |
| BmorOBP35 | Gong et al. 2009 |
| BmorOBP36 | Gong et al. 2009 |
| BmorOBP37 | Gong et al. 2009 |
| BmorOBP38 | Gong et al. 2009 |
| BmorOBP39 | Gong et al. 2009 |
| BmorOBP40 | Gong et al. 2009 |
| BmorOBP41 | Gong et al. 2009 |
| BmorOBP42 | Gong et al. 2009 |
| BmorOBP43 | Gong et al. 2009 |
| BmorOBP44 | Gong et al. 2009 |
| HvirABP1 | CAC33574 |
| HvirABP2 | CAC33575 |
| HvirABPX | CAA05508 |
| HvirGOBP1 | CAA65605 |
| HvirGOBP2 | CAA65606 |
| HvirPBP1 | CAA65604 |
| HvirPBP2 | CAL48346 |
| MsexABP1 | AAF16713 |
| MsexABP2 | AAL60416 |
| MsexABP3 | AAL60413 |
| MsexABP5 | AAL60423 |
| MsexABP6 | AAL60424 |
| MsexABP7 | AAL60425 |
| MsexABP8 | AAL60426 |
| MsexABPX | AAF16699 |
| MsexGOBP1 | AAA29315 |
| MsexGOBP2 | AAG50015 |
| MsexPBP | AAF16715 |
| MsexPBP2 | AAF16710 |
| MsexPBP3 | AAF16703 |
| PxylGOBP1 | ABY71034 |
| PxylGOBP2 | ABY71035 |
| PxylPBP1 | ACI28451 |
| PxylPBP2 | BAG71422 |
| SexiGOBP1 | ACY78412 |
| SexiGOBP2 | CAC12832 |
| SexiPBP1 | AAS46620 |
| SexiPBP2 | AAS55551 |
| SexiPBP3 | ACY78413 |
