# Supplementary material for: An Expressed Sequence Tag collection from the male antennae of the Noctuid moth Spodoptera littoralis: a resource for olfactory and pheromone detection research
Source: BMC Genomics. 2011 Jan 29;12:86. doi: 10.1186/1471-2164-12-86 (PMC3045336; doi:10.1186/1471-2164-12-86)
Supplement: Additional file 7 — Table reporting the forward and reverse primer sequences used in real-time PCR, annealing temperatures, resulting amplicon lengths and PCR efficiencies. [file 1471-2164-12-86-S7.DOC]

| **Unigene accession number** | **qPCR Forward primer sequence (5' to 3')** | **qPCR Reverse primer sequence (5' to 3')** | **Annealing T (°C)** | **Amplicon lengh (pb)** | **Efficiency** |
| --- | --- | --- | --- | --- | --- |
| EZ983476 | GCTGGGACCTTGATGAGTATTG | CACGCATTGGACGCAGTTATAG | 57 | 150 | 1.96 |
| EZ983129 | CATTCACACAAACATCCGTCAC | ACCCAGCGTACTTGTATCAAAG | 55 | 156 | 2.07 |
| EZ983645 | CACGAGTCTTCAGGAGTCATC | GCGGCAGAAGTCACATAGG | 53 | 111 | 1.94 |
| EZ982362 | GTCGCATCCTTCAGGTAGTCC | TCGGGCATAATGTCTTCAATCC | 58 | 121 | 1.84 |
| EZ981187 | GTATGGGATGCTGGTGAGAGAAG | AGTGGATTGAAGACCTGGATATGC | 58 | 163 | 1.84 |
| EZ982965 | CCGCAGACATGAGATCAGGAAC | CCATAAGACACCACCACCATCC | 58 | 156 | 1.97 |
| EZ982621 | CAGACGAAGCAGACACCAATTC | TGGGTACGCTGGGATGAGAC | 58 | 118 | 2.24 |
| EZ981394 | GTGAGTGCGAGCGTGAAGC | TGCCACACCGTCTCTACAGG | 58 | 169 | 1.87 |
| EZ981024 | CGTCATCACCCACAACCTCAC | CCCAATAGTCACCCAGCCAAAG | 57 | 196 | 1.99 |
| EZ983328 | GAAGATGAATCTGTGCAGGG | ATGAACCGATTTGGCAGCCA | 62 | 179 | 1.88 |
| EZ981960 | GCCACTGACGATAATACTGAC | GACTCCAACATTAGCAACACC | 62 | 211 | 1.88 |
| EZ983448 | TGTAACTGGCGAGGGAAATCAC | GCTCTATATGGCTGCGGTTGG | 58 | 133 | 1.80 |
| EZ982994 | CGTCATCACCCACAACCTCAC | CCCAATAGTCACCCAGCCAAAG | 58 | 196 | 1.88 |
| EZ981646 | CTGCCGCCTGCTTCTACTAC | GATGCCAAGAGACAAGACAACC | 60 | 113 | 1.87 |
| EZ981047 | CCTCTTCTTCACTCACACCATC | TAGCGGGCATCTGACTCC | 53 | 129 | 1.84 |
| EZ982777 | CGAGTTGGGTCACACTTATAT | GGAGTCAGGTTGAACAGCAAT | 60 | 249 | 1.91 |
| EZ981417 | TGCTCGTATTCGGAGTGGTATG | TTCGTGTGCCAACATATTCCTG | 55 | 131 | 1.98 |
| GW825563 | CGCCGAGGGTTTGTATTTG | TATATCTCTGTGCTCTGGTTCC | 53 | 148 | 2.00 |
| FQ031836 | TGGCTCGTGTTCGGTTTCG | TTGGTTGTGGGTAGATCAGGTC | 55 | 127 | 2.00 |
| FQ031000 | GTGTGGCAGCAGCGTATC | AACTTGATGGCAACCTCTTCC | 62 | 210 | 1.90 |
| FQ030158 | TGTTACCGATCTGTCGTTACGC | GACCTTGACGCCTCACCATC | 58 | 241 | 1.94 |
| FQ016677 | AGGTATTCAATCACGGCTTCC | TGTGTTCTGTTCGCTGGTC | 53 | 105 | 1.74 |
| FQ016760 | AAGAAGAAATTGGAGGCAGCAG | AGCATAAAGCAGAGCACAGATG | 60 | 125 | 2.00 |
| FQ017398 | ATGGGCTTCAGATTGGGTTGG | GGCAATTCCGACTTGGTCTCTC | 58 | 116 | 1.98 |
| FQ014255 | TGCGACCTGCCGACTATG | CTCCTCACGAACACGAACC | 55 | 179 | 2.28 |
| FQ015038 | TGGGAGGATCTATACGGCTTG | GCGAGTCCAACATACATCTGC | 57 | 183 | 1.84 |
| S. littoralis *RpL8* | ATGCCTGTGGGTGCTATGC | TGCCTCTGTTGCTTGATGGTA | 53/62 | 189 | 1.90 |
